# Gender Identification Beyond the Binary and Its Consequences for Social Well-Being

**DOI:** 10.1007/s10508-022-02453-x

**Published:** 2022-11-14

**Authors:** Miriam Ines Wickham, Félice van Nunspeet, Naomi Ellemers

**Affiliations:** 1 Organisational Behaviour, Faculty of Social and Behavioural Sciences, Utrecht University, 3584 CS Utrecht, The Netherlands; 2 Organisational Behaviour, Faculty of Social and Behavioural Sciences, Utrecht University, Utrecht, The Netherlands

**Keywords:** Gender identification, Gender non-conformity, Non-binary, Social well-being, Authenticity

## Abstract

Recent societal initiatives (e.g., gender-neutral toilets, clothing, and language) highlight the ongoing shift of gender away from binary categories: “man” and “woman.” We identified and investigated two reasons for this shift: that many people may not identify with strictly binary categories and that this may have negative social consequences. Employing a multiple-identification model, we measured intergroup self-categorization with both men and women (Studies 1 and 2), as well as with a “third gender” (Study 3) and investigated how multiple identifications are related to social well-being (Studies 2 and 3). In Study 1 (*N* = 182, mean age = 32.74, 121 women), we found that a binary model was not the best fit for our gender identification data. In Study 2 (*N* = 482, mean age = 30.98, 240 AFABs), we found four clusters of gender identification, replicating previous research. Furthermore, we found that gender non-conforming participants reported being less able to be their authentic selves than binary participants. We also found that participants who identified lowly with both binary genders reported lower well-being in general (belongingness, self-esteem, life satisfaction, positive affect). In Study 3 (*N* = 280, mean age = 36.97, 140 AFABs), we found that asking about a third gender seemed to change how much participants reported identifying with men and women. We also found that gender non-conforming participants reported lower authenticity, belongingness, and self-esteem. We conclude that moving away from binary categories of gender may be beneficial to many non-conforming people of different nationalities, including cisgender, heterosexual people.

**Supplementary Information:**

The online version contains supplementary material available at 10.1007/s10508-022-02453-x.

## Introduction

Throughout the developed world, there has been an increase in legal recognition of a “third” gender/sex category, which is separate from male or female (e.g., in Australia: Bennett, 2014; in Germany: Dunne & Mulder, 2018). The legal recognition of more than two genders (e.g., “man,” “woman” and “non-binary”) is an example of the current shift away from the “gender binary,” where there are only two gender categories, and toward a “gender spectrum,” to acknowledge that there are multiple ways for people to define their gender. Recent estimates suggest that anywhere from 3 to 35% of the general population see themselves as part man and part woman, or as something completely different from man and woman (in the Netherlands: Kuyper & Wijsen, 2014; in Flanders, Belgium: Van Caenegem et al., 2015; in Israel: Joel et al., 2014). We set out to further examine these different options to indicate gender, by allowing more freedom for expression of gender identification (GI) in our questionnaire items. We suspected that this would reveal a larger percentage of gender non-conformity in the general population than some of the previous work. Furthermore, we argue that being gender non-conforming in a society with a binary narrative would have negative consequences for one’s social well-being. Across three studies we therefore tested the implications of gender non-conformity on social well-being, specifically, societal inclusion, general self-esteem, and life satisfaction.

### Toward a More Inclusive Gender Conceptualization

Our world is highly binary in terms of gender. The gender binary is learnt from a young age, for instance, when teachers implicitly treat boys and girls differently (e.g., Duffy et al., 2001; Tsouroufli, 2002), and binary gender labels on toys affect children’s play behavior (Yeung & Wong, 2018). When asked to indicate gender in registration of person characteristics, most forms provide only two options (Morgenroth & Ryan, 2018) and the binary gender categories are (sometimes falsely) considered biologically essential and meaningful (Skewes et al., 2018). In social psychology, research into GI is a little more nuanced, in that participants are generally asked to state the extent to which they identify with their binary gender group, based on which we classify low or high identifiers (e.g., Derks et al., 2011). However, such an approach is still binary because a participant of the female sex is asked only to indicate how much they identify with women, thereby disregarding other-sex GI. This is an example of how most of the psychological literature regards gender identification as a binary construct, though not all, as will be explained.

Importantly, a large body of research has investigated psychological androgyny in terms of personality traits. This research began with Bem (1974) and has impacted gender research greatly, by promoting equality between men and women, showing the malleability of gender roles, and underlining the negative effects of polarization of gender groups (Dean & Tate, 2017). This body of work was an important steppingstone for research into gender beyond the restrictive, binary conceptualization that is still prevalent today. The foci of this research were gender roles, as measured through personality traits. For instance, women are typically stereotyped to be caring and tender, while men are typically thought of as agentic and dominant, and these stereotypes have persisted across decades (Bem, 1974; Haines & Stroessner, 2019). The present paper was inspired by this important literature, but our focus is on gender identification, meaning self-categorization as a member of different social (gender) groups (Turner & Reynolds, 2011). While gender roles are about the way that people are stereotyped in terms of personality traits, gender identification is about feelings of group membership. We also show that these two concepts are distinct in our own data; the results of this are beyond the scope of this paper, but can be found in the Supporting Information.

The idea that gender is a binary construct is currently changing. We can see this in the legal recognition of non-binary genders (e.g., in Australia: Bennett, 2014; in Germany: Dunne & Mulder, 2018), and in the increase of gender-inclusive interventions, such as gender-neutral toilets (Gershenson, 2010), unisex clothing lines (Park, 2014), and gender-neutral language (Hord, 2016), among others. In this paper, we identified two possible reasons for this change. First, people may feel that the gender binary does not reflect the way they see their own gender. Second, the gender binary restricts people in such a way that their well-being is compromised. As such, we tested gender identification (in terms of intergroup self-categorization) in the general population, and how this relates to social well-being. We describe each of these research questions below.

### Gender Identification Beyond the Binary

While one might assume that most cisgender, heterosexual (i.e., normative) individuals have much less complex gender identities than people belonging to the LGBTQIA + conglomerate, research suggests that this is not the case. This means that the gender binary may not be a good reflection of the way people in the general population identify. Cisgender, heterosexual participants in an interview study showed much diversity in how they perceived their own gender and sex (Abed et al., 2019). Various studies have employed a multiple-identification model of gender and found that both normative and non-normative individuals can identify with various genders at once. A multiple-identification model is one in which participants, regardless of their explicit social category (e.g., “woman”), are asked how much they identify with several categories (e.g., “man” and “woman”) of the same construct (e.g., gender). For instance, Joel et al. (2014) asked Israeli participants how much they felt like a woman, a man, both or neither in the past twelve months. They found that some (self-assigned) men felt more like a woman than a man, and vice versa, and that approximately 35% of men and women felt like both a man and a woman. Similarly, Martin et al. (2017) asked US children (ages 5–10 years; whose gender identity and sexuality is not fully formed yet) how similar they felt to members of their own assigned gender at birth (AGAB) as well as how similar they felt to members of the “other” gender. They found that children could be clustered into four distinct categories: Own-Gender Similar (high identification only with own AGAB), Cross-Gender Similar (high identification only with “other” gender), Both-Gender Similar (high identification with both binary genders) and Low-Gender Similar (low identification with both binary genders). They estimated that 30% of children were Both-Gender Similar. Various studies have used Martin et al.’s (2017) gender similarity measure and largely replicated their results in Dutch, Italian, and US samples of young adults (Andrews et al., 2019; Baiocco et al., 2021; Endendijk et al., 2019). Similarly, Pauletti et al. (2017) found relatively low correlations between own-gender typicality and other-gender typicality in children, further showing that these are two distinct concepts.

Similarly, using a multiple-identification model, there is a small body of literature examining so-called gender ambivalence within the Dutch and Flemish context. Gender ambivalence is a term coined by Kuyper and Wijsen (2014) to describe identification that is approximately equal for both binary gender groups. A gender ambivalent person therefore identifies with women to a similar extent as they identify with men. Kuyper and Wijsen (2014) and Van Caenegem et al. (2015) estimated that in the Netherlands and Belgium, respectively, 3–5% of people are gender ambivalent. Gender ambivalence is a form of “gender non-conformity,” the term we use throughout this paper.

In the present paper, we tackled the research question of how both cisgender and heterosexual, as well as LGBTQIA + adults identify in terms of their gender, by combining methods from these four previous studies, and adding to them. Previous papers differed from each other in various aspects, and in this section, we address each of these aspects, and describe how we tackled them in our own studies.

While most of the previous studies each included one measure of social gender identification, consisting of one item (in Joel et al., 2014, “feeling like a man/woman;” in Kuyper & Wijsen, 2014, and Van Caenegem et al., 2015, “experiencing oneself as a man/woman”), Martin et al. (2017) and Pauletti et al. (2017) measured gender identification using scales including several items. We argue that to measure a complex a concept as gender identification, one must allow the participant to reflect on various aspects of their gender, by including several items that capture the diverse ways in which participants may feel about their gender.

Moreover, in one study identification with “women,” “men,” “both,” and “neither” was each measured using separate items (Joel et al., 2014). However, most other studies measured identification with “women,” and with “men,” and inferred which participants identified with both or neither from the data (e.g., those participants scoring low on identification with women and low on identification with men, were considered to identify with neither; Andrews et al., 2019; Baiocco et al., 2021; Endendijk et al., 2019; Kuyper & Wijsen, 2014; Martin et al., 2017; Pauletti et al., 2017; Van Caenegem et al., 2015). We argue that either method is valid for assessing identification with “both” and “neither” men and/nor women, however, we favor simplifying the participant’s experience in answering questions where possible. Consistent with work by Kuyper and Wijsen (2014), Martin et al. (2017), and Van Caenegem et al. (2015), we thus measure identification with “men” and with “women” (and a third gender in Study 3), and do not additionally ask participants to indicate how much they identify with “both” or “neither.”

Furthermore, in previous studies, identification was measured in relation to binary groups (“men” and “women”), and genders outside of the binary were not included in the model. Such a model is less binary than other work, where participants are asked about only one binary gender group. However, since all questions relate back to the binary gender groups it implies that one cannot identify with a gender that is unrelated to men and women. We argue that it is important to explore how participants’ responses may differ if asked only about binary genders, or about binary and non-binary genders.

Each of these former studies also used different methods, in line with their measures, for calculating estimates of the prevalence of gender non-conformity, or the amount of people who identify with multiple gender identities (e.g., in Martin et al., 2017, a cluster analysis; in Joel et al., 2014, frequencies divided by gender group; in Kuyper & Wijsen, 2014, and Van Caenegem et al., 2015, descriptive statistics across the whole sample). Given that we argue for more complex questionnaires with diversity in items for measuring identification, we favor a cluster analysis, which allows us to display the complexity of the data in a simplified manner for the reader. This methodology may seem reminiscent of Bem’s (1974) approach to measuring and scoring psychological androgyny. This approach displayed masculinity and femininity as orthogonal dimensions rather than a linear construct with two endpoints and categorized people into groups (e.g., psychologically androgynous) according to their femininity and masculinity scores (or rather, the difference score between them). We similarly argue that gender identification with various gender groups should be seen as orthogonal and not a linear construct with two endpoints. Using cluster analyses, gender identification scores with various gender groups can be seen as statistically independent. We clustered participants into groups based on those scores for the purposes of giving estimates and conducting further analyses only, and not to claim that the clusters we find should be seen as the new norm for categorizing gender. However, we also argue that cross-validation of measures is important, to show that similar results can be obtained, regardless of the method used.

Lastly, in each study the participants shared a country of residence: the USA (Andrews et al., 2019; Martin et al., 2017; Pauletti et al., 2017), Israel (Joel et al., 2014), Italy (Baiocco et al., 2021) the Netherlands (Endendijk et al., 2019; Kuyper & Wijsen, 2014), and Belgium (Van Caenegem et al., 2015). The redefinition of gender as a non-binary construct is happening world-wide, and to compare whether participants in different studies experience their genders in similar ways, a multinational sample is necessary.

### Reduced Well-Being

Importantly, in previous work, there has been less of a focus on the relationship between gender non-conformity and well-being. Social identification, including gender, affects social well-being because some social categories are more discriminated against than others. Since the societal norm is that “gender is binary” and this is perpetuated from a young age, it is likely that people consider gender non-conformity to be an undesirable or uncommon trait, which would have negative consequences for gender non-conforming people. For example, it has been found that people feel negatively about physically androgynous people, who are difficult to categorize in a binary way (e.g., Stern & Rule, 2018). A highly binary society also means little representation of gender non-conformity in politics or media, which can cause negative feelings in gender non-conforming people (Klasen, 2007). Gender non-conforming people may feel invisibly dissimilar from other people, whom they assume to be binary, which can reduce feelings of inclusion (Şahin et al., 2019). Because of the perceived deviance from the social norm, we expected that gender non-conforming individuals would report lower social well-being. Such a finding would underline the need to move away from binary conceptualizations of gender in society.

We were interested in social well-being, rather than clinical well-being. Examinations of clinical symptoms, such as gender dysphoria, depression, and anxiety, in people who identify with a non-normative gender, can be found in earlier work (e.g., Dhejne et al., 2016; Kuyper & Wijsen, 2014). This is important work, as it shows that there are serious clinical implications to non-normative gender identity. However, one does not have to have a clinical diagnosis to be suffering from the consequences of being non-normative in a binary society. People with non-normative gender identifications may feel less included by society, because they feel dissimilar from the social norm (Kristof-Brown et al., 2005). Specifically, they may feel that they are not allowed to be their authentic (non-normative) selves, or that they do not belong with other (binary) people (Jansen et al., 2014). Because their personal identification does not fit with the collective, people may have lower personal self-esteem, meaning lower acceptance of one’s own personal identification (Rosenberg, 1979). They may also feel that their general quality of life is impacted by their lowered inclusion, meaning they are less satisfied with their lives (Diener et al., 1985). Lastly, their emotional profile might be impacted negatively, without necessarily fitting the diagnostic criteria for a mood disorder, such that they feel more negative affect and less positive affect in their lives (Andrews & Withey, 1976). All of these measures are related to one another and are part of well-being as a member of a social environment. In our studies, we measured social well-being in terms of feelings of inclusion in society as well as general self-esteem, life satisfaction, and positive and negative affect, and hypothesized that we would find differences between gender conforming and gender non-conforming participants in all of these measures.

Social well-being, while being affected by one’s (private, internal) gender identification, is also affected by how one is categorized by others (externally). One may identify as both man and woman but be perceived solely as a man. This is related to biological features that are perceived as male or female, which tend to correlate with one’s assigned sex at birth (ASAB). It can also be related to how one expresses themselves, for instance one’s name, clothes, or pronouns, which tends to correlate with one’s self-assigned gender identity. Men tend to have more status in society than women (e.g., Eagly, 1983), and therefore feel more included by society in general. As such, being perceived as a man may increase feelings of social well-being. Self-assigned gender identity and ASAB are, therefore, potential moderators for the relationship between gender (ambivalent) identification and social well-being, which we tested.

### The Present Studies

In our studies, we estimated the prevalence of gender non-conformity across three multinational samples by employing a multiple-identification model, and using a questionnaire that measures self-categorization as a member of a social group. We hypothesized that we would find four clusters of GI, in line with Martin et al. (2017). We hypothesized that at least one of those clusters could be considered gender non-conforming (in Studies 1 and 2, identifying the same amount with both gender groups; in Study 3, identifying to a certain extent with a third gender). By using this methodology, we expected to find a higher percentage of gender non-conforming people than previous estimates reported by Kuyper and Wijsen (2014) and Van Caenegem et al. (2015).

We measured intergroup self-categorization (Turner & Reynolds, 2011), which is the cognitive component of social identification (Ellemers et al., 1999), using a scale that encompasses various aspects of self-categorization: identification as a member of the group, collective self-esteem (i.e., how important a group membership is to the self), and similarity to the group. We argue that using such a scale provides the participant with freedom to reflect on various parts of their gender self-categorization. We also argue that, while self-categorization theory (Turner & Reynolds, 2011) poses that individuals define themselves as members of distinct social groups, in the case of gender, the binary groups of “men” and “women” may not be entirely distinct, having somewhat blurry social boundaries. By asking people how much they self-categorize as men and women, we aim to show that participants acknowledge these blurry social boundaries and can identify with both, with neither, or with some other gender entirely. Additionally, to cross-validate our gender self-categorization scale, which relies on participants’ understanding of language, we compared the results of the scale with the results of an alternative, pictorial (i.e., less reliant on language) measure of self-categorization, which we included in Study 3.

To test our prediction that gender non-conformity, as compared to binary identification, would be related to lower social well-being, in Studies 2 and 3 we included measures for societal inclusion, general life-satisfaction and self-esteem. In Study 2 we additionally measured positive and negative affect when reflecting on those measures. We also tested the potential moderation effect of ASAB and self-assigned gender identity on the relationship between gender non-conformity and social well-being.

## Study 1

### Method

#### Participants

Based on a medium effect size (approx. Cohen’s *f* = 0.25), as is typical in social psychology in general (Richard et al., 2003), and the expected number of GI categories being four, we calculated that our *N* would need to equal at least 179 participants to achieve 80% power.^1^ We recruited 182 participants, whose age ranged from 18 to 64 (*M* = 32.74, *SD* = 10.05). Sixty participants said that their gender was male; 121 participants said they were female, and one person indicated being “Other.” Our sample was predominantly living in the United Kingdom (55.9%), as is usual on Prolific, with the rest of participants spread out across eighteen other (mostly Westernized) countries world-wide. For (more) information about participants’ country of residence, education level, employment situations, level of feminist identification or LGBT + identification, please see the Supporting Information. It should be noted that the results of this study are not affected by any of these demographic profiles.^2^

#### Materials

##### Gender Identification

To test the extent of self-categorization as each gender group, seven items were administered about each gender group.^3^ Items included “I identify with (other) women/men” and “I feel that I belong to the group of women/men” (for all items, please see Supporting Information). All items were administered twice, with the items being identical in both cases, other than the replacement of the word “men” with “women” and vice versa (items about men α = .86, items about women α = .91; for factor analysis, see Supporting Information). They were answered on a Likert scale going from 1 (highly disagree) to 7 (highly agree). For gender identification with women, we took the mean of the items about women, while for gender identification with men, we took the mean of the items about men. We used mean scores, rather than individual items, in order to be able to treat these as continuous variables, and to be able to measure more variance in gender identification.

#### Procedure

Participants were recruited using the online platform Prolific. They were redirected from Prolific to the survey’s Qualtrics link and asked to answer all questions. Participants were first asked about their gender identification, with items shown in a random order. They were additionally asked to answer questions of the following scales, which are beyond the scope of the current paper^4^: Bem Sex Role Inventory, two questionnaires about typically feminine and typically masculine norms, belief in “gender-as-binary” or “gender-as-spectrum” questions. Lastly, they were asked demographic questions. They were shown a debriefing and given the opportunity to retract their data. Participants received payment for their participation.

## Results

### Gender Identification

We created two variables: gender identification with women (GIW) and gender identification with men (GIM). We found a significant difference in GIM and GIW; respectively *M* = 3.64, S*.D*. = 1.28, min. = 1.00, max. = 7.00; *M* = 4.29, *S.D.* = 1.37, min*.* = 1.00, max. = 7.00; *t*(181) = 4.24, *p* < .01, Cohen’s *d* = 0.29; indicating that our sample on the whole had a higher GIW than GIM. This may be due to the higher number of female participants in the sample.

### Relationship Between Gender Identification with Women and Gender Identification with Men

**Fig. 1:**
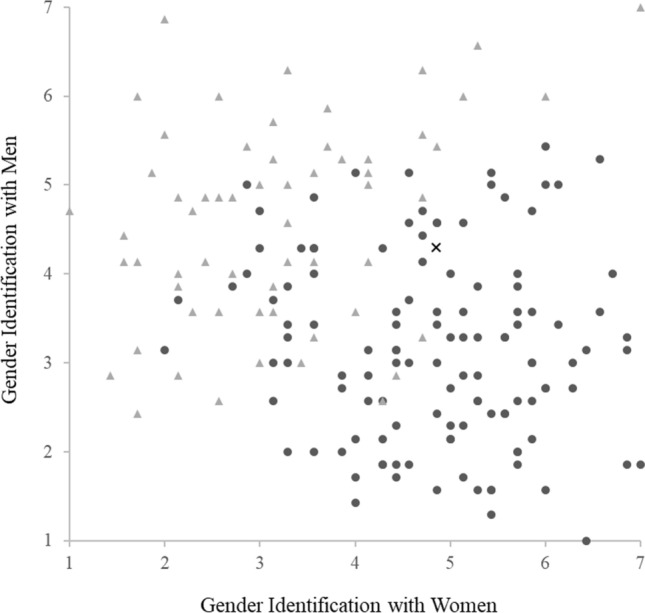
Scatter plot of gender identification results from Study 1 *Note.* Triangles represent self-reported men, circles represent self-reported women, and the X represents the self-reported Other participant

While we established that our GIW items loaded onto a different factor than our GIM items, thereby suggesting that they are two different concepts, we wanted to test how much they correlated with each other. While two concepts can be distinguishable from one another, they may still be highly related. In the case where GI is a spectrum with two opposite ends (male and female), one would expect that GIM would be moderately to highly negatively correlated with GIW. To test this we ran a Pearson’s correlation which revealed only a small negative correlation between GIM and GIW (*r*(180) = − .26, *p* < .01, see Fig. 1). This indicates that, while GIW is negatively correlated with GIM, they cannot be considered complete opposite ends of the same spectrum.^5^

Given the low correlation between GIM and GIW, we wanted to test how many clusters of GI participants would fall into. To test this, two-step cluster analyses with two inputs (GIM and GIW) and different numbers of clusters (two, three, four and five) were run. In terms of silhouette of cohesion and separation, the qualities of the 2-cluster, the 3-cluster and the 4-cluster model, but not the 5-cluster model, were all similar and good (silhouette  ≥ .5) Most importantly, Bayesian Information Criteria of the different cluster models (BICs, used to compare different models with each other), showed that splitting the participants up into three clusters resulted in better (lower) BICs than the 2-, 4- or 5-cluster models; 3-cluster model *BIC* = 174.68, *BIC* change = 23.03. Thus, a binary model of gender is not the best fit, and participants could best be categorized into three GI categories. This is one less than hypothesized based on previous literature (Martin et al., 2017). Based on the cluster centers (see Table 1), we suggest that the missing cluster is a low male, low female identifying cluster.

**Table 1:** Cluster centers for gender identification with women and gender identification with men in Study 1

	Cluster 1 (high male, low female identifiers)	Cluster 2 (high male, high female identifiers)	Cluster 3 (high female, low male identifiers)
Cluster center for GIW	2.74	5.04	5.13
Cluster center for GIM	4.18	4.95	2.56

Absolute range of GIM and GIW: 1–7

## Discussion

Our goals for the first study were (1) To test whether a binary or dual-identification model of gender fit the adult people’s identifications better, and (2) To add to the literature about gender non-conformity by providing another estimate of the occurrence of gender non-conformity, using our methods.

We found that a dual-identification model of gender fits our data better than a binary model of gender. Specifically, we found three clusters of GI, one less than previously found by Martin et al. (2017) in children. Martin et al. (2017) had found that the four gender clusters were male identifiers, female identifiers, identifiers of both, and identifiers of neither (low male as well as low female identification). It seems that, in our data, we did not find low identifiers of both genders. This may be due to the development of GI between childhood and adulthood, but we explore this further in Study 2.

A limitation to this study was the overrepresentation of women over men, which caused us to refrain from providing an estimate of the occurrence of gender non-conformity as this would likely be skewed. To circumvent that in Study 2, we recruited equal numbers of people assigned male and female at birth. As such, a main aim of Study 2 was to report percentages of people in each cluster of the model, given a sex-equal sample.

Furthermore, our sample in Study 1 was mostly living in the UK or in the Westernized world, while changes in the gender narrative are happening world-wide. We therefore wanted to recruit a more international sample in Study 2 in terms of country of residence.

Lastly, we were interested in the relationship between GI and social well-being. In Study 1, we had not included any measure of social well-being. As such, in Study 2, we measured well-being, more specifically, in terms of societal inclusion (authenticity and belonging), general self-esteem, life satisfaction and affect (positive and negative). Our hypothesis was that gender non-conformity would be related to lower feelings of well-being because the normative system is the gender binary, and gender non-conforming people may not feel that they “fit” into the normative system. We also explored some potential mediators and moderators for the relationship between GI and well-being, namely ASAB, and self-assigned gender identity.

## Study 2

### Method

#### Participants

Based on the small effect sizes found in Study 1 and based on previous literature (Goldman & Kernis, 2002; Richard et al., 2003) we expected our effect sizes to be medium-small (approx. *f* = 0.15). Based on Study 1, we expected to find three GI categories, however, because of the underrepresentation of men in that sample, we kept the possibility of finding four groups (like Martin et al., 2017) open. We would run ANOVAs to measure differences in six measures of well-being. Given this, we calculated a required *N* of 450 participants to achieve 80% power.^6^ We recruited 482 participants with an equal number of AMABs and AFABs (*N* = 241 each), using Prolific’s pre-screening feature (participants were asked the following question: “What sex were you assigned at birth, such as on an original birth certificate?”). A total of 242 participants identified as men, 240 identified as women, and none identified as other. Participants’ age ranged from 18 to 76 (*M* = 30.98, *SD* = 9.94). Additionally, since our sample in Study 1 was predominantly White, while the inclusion of non-binary genders is a phenomenon that crosses the boundaries of ethnic privilege, we also took care to recruit an equal number of White and non-White participants (*N* = 241 each). This was also done using Prolific’s pre-screening (participants were asked the following question: “What is your ethnicity?”^7^). Our sample in Study 2 was also more diverse in terms of country of residence than in Study 1. Participants lived in 36 different countries world-wide (not all of which Westernized), with a majority living in the UK (38.40%) and the US (19.71%). For more information about participants’ ethnicity, education, employment, special needs, and country of residence, see Supporting Information. We also controlled for these demographics and, as in Study 1, did not find that any of them affected the results reported in this manuscript.

#### Materials

##### Gender Identification

We used the gender identification scale from Study 1 but had to exclude four items (two about men and two about women) due to high cross-factor loadings (see Supporting Information).^8^ We therefore measured GI using 10 items (five about men, α = .92; five about women, α = .90).

#### Social Well-Being

##### Inclusion Scale

We administered Jansen et al.’s (2014) Inclusion scale developed to assess organizational inclusion and adapted the items to refer to “members of my society.” There were 16 items; they included “Members of my society give me the feeling that I belong” and “Members of my society allow me to be authentic.” Eight of the items were about belongingness (α = .97); the other eight were about authenticity (α = .96).

##### Self-Esteem

To measure general self-esteem, we administered 10 items which included “On the whole, I am satisfied with myself” and “At times I think I am no good at all” (reverse coded; Rosenberg, 1979; α = .92).

##### Life Satisfaction

To measure general life satisfaction, we administered five items which included “In most ways my life is close to my ideal” and “If I could live my life over, I would change almost nothing” (Diener et al., 1985; α = .85).

##### Positive and Negative Affect Schedule

This scale consists of 20 emotion words, 10 of which are positively valenced (e.g., “inspired”) and 10 are negative (e.g., “ashamed;” Watson et al., 1988). We asked participants to reflect on how they felt while answering the questions about their gender, societal inclusion and well-being, and to rate how much they felt a certain emotion from the Positive and Negative Affect Schedule (PANAS) during that time (positive items α = .88, negative items α = .93).

#### Procedure

Participants were recruited through Prolific, using restrictions to recruit equal numbers of people in terms of ASAB and ethnicity. They were redirected from Prolific to the survey’s Qualtrics link and asked to answer all questions. First they were asked to state their self-assigned gender. Next, they answered questions about GI, followed by the societal inclusion questionnaire, followed by the self-esteem, life satisfaction and emotion questionnaires. There were several extra measures that were administered to participants, the data of which we do not report in the paper as it is beyond the scope of this article: need to belong to ethnic and gender groups, beliefs about gender as a construct, and questions about a recent BBC article (see Supporting Information). The items within each questionnaire were administered in a random order. Last, participants answered demographic questions. They read a debriefing and had a chance to retract their data from the study. Participants received payment for their participation.

## Results

### Gender Identification

**Fig. 2:**
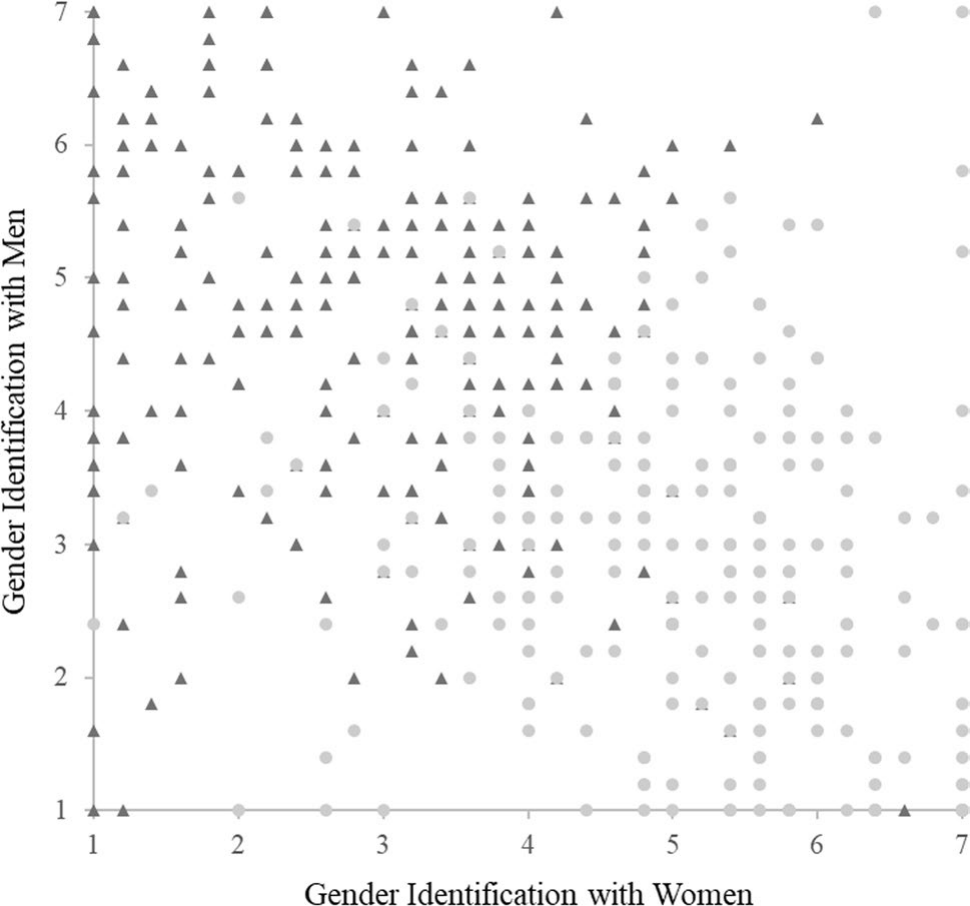
Scatter plot of gender identification results from Study 2 *Note.* Triangles represent participants assigned male at birth, circles represent participants assigned female at birth

Mean scores of GIW and GIM did not significantly differ from one another; *t*(481) = 1.27, *p* = .21, *M* of GIW = 3.96, *M* of GIM = 3.80. A Pearson’s correlation revealed that the correlation between GIM and GIW was small to moderate and negative (*r*(480) = − .47, *p* < .01, see Fig. 2).

Several two-step cluster analysis with two inputs (GIW and GIM) revealed that a 4-cluster model (as compared to 2, 3, 5 and 6-cluster models) was the best fit in terms of percentage of variance, Bayesian Information Criterion (*BIC* = 340.54) and silhouette of cohesion (silhouette ≥ .5) and separation combined. This is different from the 3-cluster model found in Study 1. A four-cluster model was therefore chosen and the cluster membership of each participant was saved in order to perform further analyses. The clusters can be translated into: (1) Male identifiers, (2) Low identifiers (who identify lowly with both binary genders), (3) Dual identifiers (who identify relatively highly with both binary genders), and (4) Female identifiers. Table 2 shows the cluster centers as well as percentages of overall sample that were placed in each cluster. It seems that the cluster which was not found in Study 1 (low identifiers of both genders) could be found in the more sex-equal dataset of Study 2, in line with previous research (Martin et al., 2017).

**Table 2:** Cluster centers for gender identification with women and gender identification with men, and percentage of participants who fell into each cluster in Study 2

	Cluster 1 (male identifiers)	Cluster 2 (low identifiers)	Cluster 3 (dual identifiers)	Cluster 4 (female identifiers)
Cluster center for GIW	2.32	2.74	4.69	5.73
Cluster center for GIM	5.52	2.93	4.42	1.99
Percentage of overall sample	25.31%	19.92%	30.08%	24.69%

Absolute range of GIM and GIW: 1–7

Given our multinational sample, and that gender is culturally constructed (e.g., Newman, 1995), we wanted to test whether participants with different countries of residence differed in their cluster memberships. A chi square test revealed no significant differences (*χ*^*2*^ = 117.82, *p* = .19), suggesting that proportions of people who are gender non-conforming do not differ per country.

### Effect of Gender Identification on Social Well-Being

We wanted to test whether GI cluster membership is related to a person’s social well-being, and whether this is moderated by self-assigned gender category and/or ASAB. We therefore ran a MANOVA with GI cluster membership (4 levels) as IV, self-assigned gender category and ASAB as moderators, and the following DVs: mean feelings of authenticity and belongingness in society, self-esteem, life satisfaction, and positive and negative affect towards answering previous questions.

As expected, we found a main effect of GI cluster membership on all measures of social well-being, except negative affect, which did not fit our prediction; authenticity, *F*(3, 478) = 20.96, *p* < .01, η_**p**_^2^ = 0.12; belongingness, *F*(3, 478) = 13.21, *p* < .01, η_**p**_^2^ = 0.08; self-esteem, *F*(3, 478) = 6.18, *p* < .01, η_**p**_^2^ = 0.04; life satisfaction, *F*(3, 478) = 6.05, *p* < .01, η_**p**_^2^ = 0.04; positive affect, *F*(3, 478) = 13.23, *p* < .01, η_**p**_^2^ = 0.08; negative affect, *F*(3, 478) = 2.56, *p* = .06). However, we did not find a main, or interaction effects, for self-assigned gender category, nor for ASAB (all *p*s > .05), contrary to expectation. To investigate which clusters differed from each other in terms of the different measures of social well-being, we ran Tukey HSD tests, which report separately for each measure, below.

#### Authenticity

**Fig. 3:**
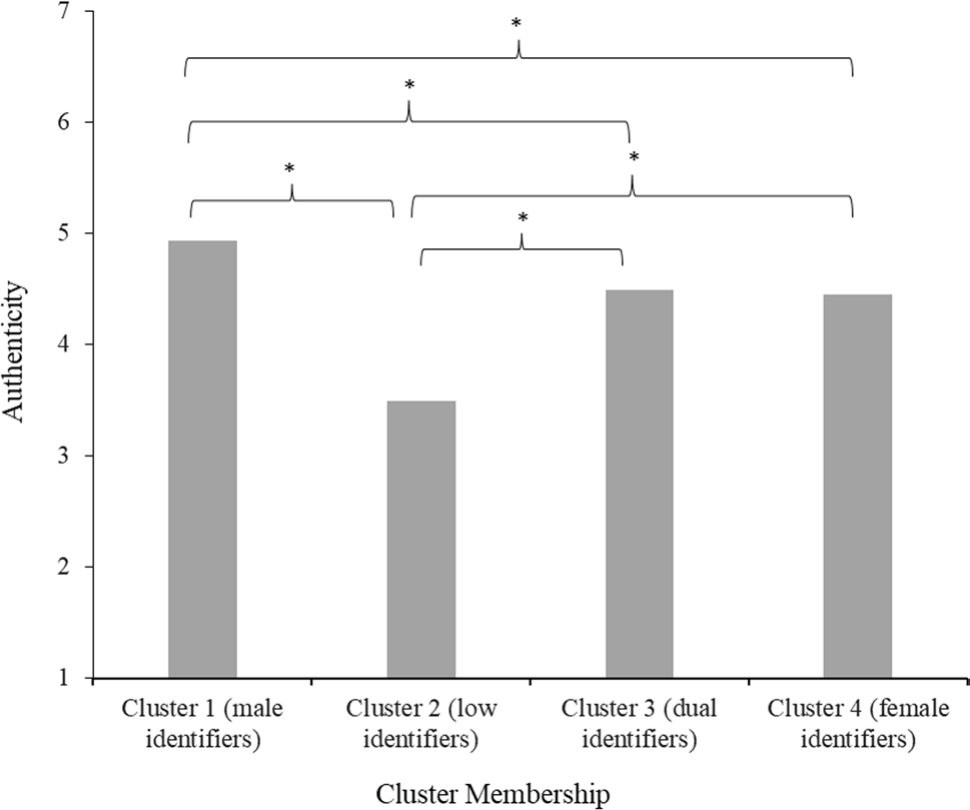
Mean feelings of authenticity in society per gender identification cluster in Study 2 *Note.* Stars (*) denote significant differences between clusters (*p* < 0.05)

The Tukey HSD test indicated that people in cluster 1 (male identifiers; *M* = 4.93, *SD* = 1.29) felt significantly more authentic (all *p*s ≤ .026) than people in all other clusters (2, 3 and 4). People in cluster 2 (low identifiers; *M* = 3.49; *SD* = 1.52) felt significantly less authentic than people in all other clusters (1, 3 and 4). Clusters 3 (dual identifiers; *M* = 4.49, *SD* = 1.24) and 4 (female identifiers; *M* = 4.45, *SD* = 1.47) did not differ in authenticity (see Fig. 3).

#### Belongingness

**Fig. 4:**
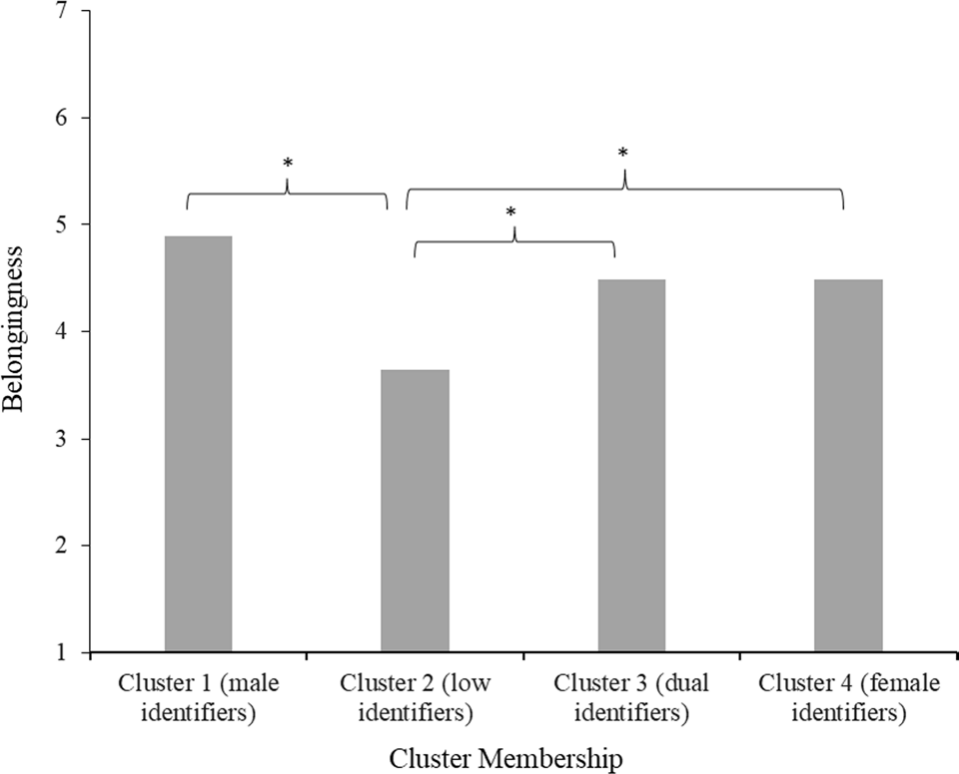
Mean feelings of belongingness in society per gender identification cluster in Study 2 *Note*. Stars (*) denote significant differences between clusters (*p* < 0.05)

The Tukey HSD test indicated that people in cluster 2 (low identifiers; *M* = 3.64, *SD* = 1.62) felt significantly less societal belongingness (all *p*s < .001) than people in all other clusters (1, 3 and 4). Clusters 1 (male identifiers; *M* = 4.89, *SD* = 1.36), 3 (dual identifiers; *M* = 4.49, *SD* = 1.35) and 4 (female identifiers; *M* = 4.48, *SD* = 1.61) did not differ from each other in societal belongingness (see Fig. 4).

#### Self-Esteem

**Fig. 5:**
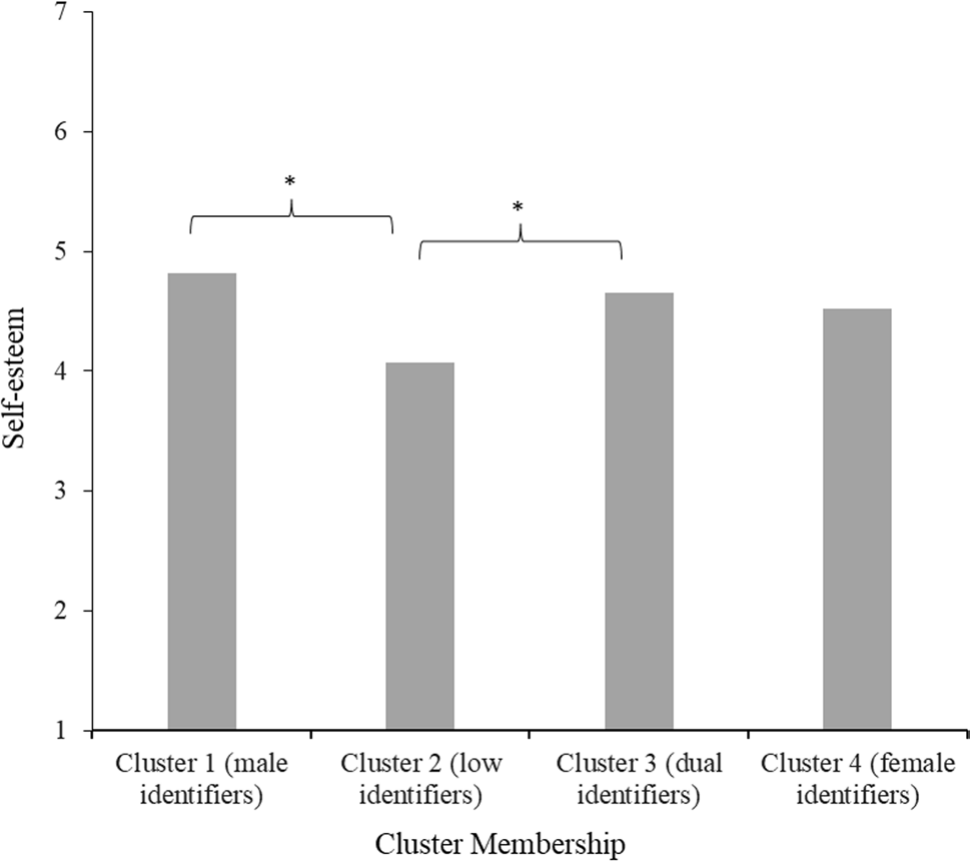
Mean feelings of self-esteem per gender identification cluster in Study 2 *Note*. Stars (*) denote significant differences between clusters (*p* < 0.05)

The Tukey HSD test indicated that people in cluster 2 (low identifiers; *M* = 4.07, *SD* = 1.43) felt significantly lower self-esteem (all *p*s ≤ .005) than people in clusters 1 (male identifiers; *M* = 4.82, *SD* = 1.39) and 3 (dual identifiers; *M* = 4.65, *SD* = 1.23), but not 4 (female identifiers; *M* = 4.52, *SD* = 1.27). Clusters 1 (male identifiers) and 3 (dual identifiers) did not differ from each other in self-esteem (see Fig. 5).

#### Life Satisfaction

**Fig. 6:**
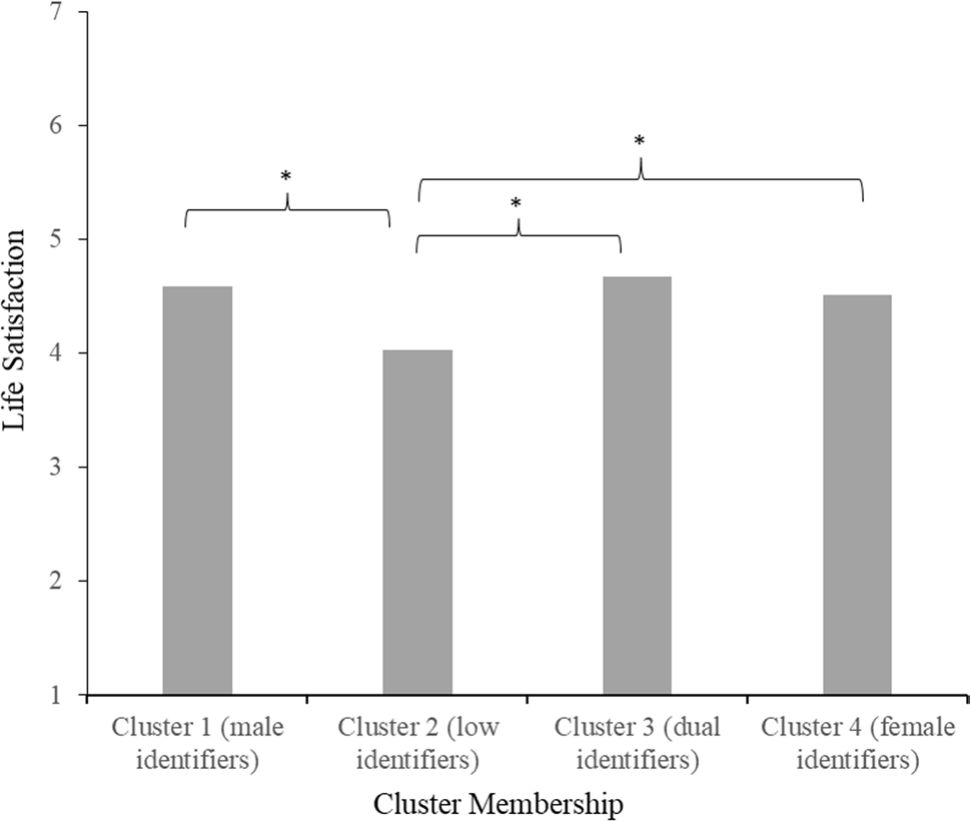
Mean feelings of life satisfaction per gender identification cluster in Study 2 *Note*. Stars (*) denote significant differences between clusters (*p* < 0.05)

The Tukey HSD test indicated that people in cluster 2 (low identifiers; *M* = 4.03, *SD* = 1.31) felt significantly less satisfied with their lives (all *p*s ≤ .020) than people in all other clusters (1, 3 and 4). Clusters 1 (male identifiers; *M* = 4.59, *SD* = 1.26), 3 (dual identifiers; *M* = 4.67, *SD* = 1.06) and 4 (female identifiers; *M* = 4.51, *SD* = 1.16) did not differ from each other in life satisfaction (see Fig. 6).

#### Positive Affect

**Fig. 7:**
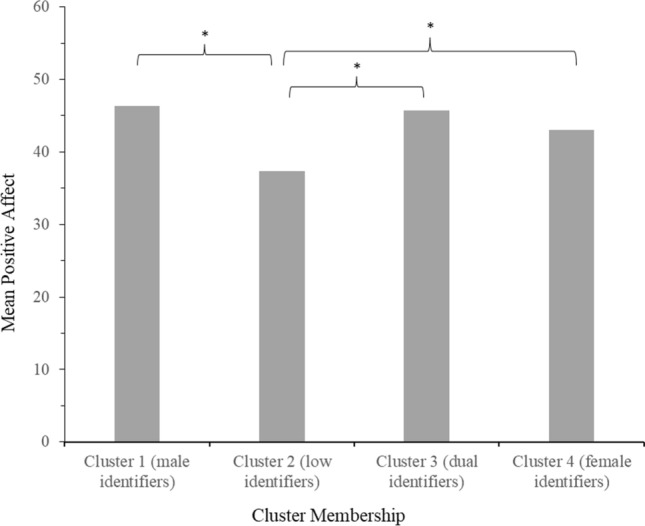
Mean feelings of positive affect per gender identification cluster in Study 2 *Note*. Stars (*) denote significant differences between clusters (*p* < 0.05)

The Tukey HSD test indicated that people in cluster 2 (low identifiers; *M* = 37.37, *SD* = 13.14) felt significantly lower positive affect (all *p*s ≤ .002) than people in all other clusters (1, 3 and 4). Clusters 1 (male identifiers; *M* = 46.36, *SD* = 11.19), 3 (dual identifiers; *M* = 45.77, *SD* = 11.12) and 4 (female identifiers; *M* = 47.07, *SD* = 11.44) did not differ from each other in positive affect (see Fig. 7).

To see whether our measures of social well-being were related to one another, we ran a Pearson’s correlation and found that all above measures were significantly correlated with one another (see Table 3).

**Table 3:** Correlations between social well-being measures, which we found to differ between clusters, in Study 2

Measure	Authenticity	Belongingness	Self-Esteem	Life Satisfaction
Belongingness	*r*(480) = .89 *p* < .001			
Self-Esteem	*r*(480) = .59 *p* < .001	*r*(480) = .50 *p* < .001		
Life Satisfaction	*r*(480) = .59 *p* < .001	*r*(480) = .52 *p* < .001	*r*(480) = .71 *p* < .001	
Positive affect	*r*(480) = .48 *p* < .001	*r*(480) = .42 *p* < .001	*r*(480) = .52 *p* < .001	*r*(480) = .50 *p* < .001

### Differences in Gender Identification Depending on Assigned Sex at Birth

To explore the relationship between ASAB and GI cluster membership, we ran a chi-square test with ASAB as IV and cluster membership as DV. It revealed a significant difference in cluster memberships (*χ*^*2*^ = 194.23, *p* < .01) across AMABs and AFABs. A post-hoc *z*-score test revealed that the differences driving the significant effect were those in clusters 1, 3, and 4: AMABs more often fell into cluster 1 (male identifiers) than AFABs, AFABs more often fell into cluster 3 (dual identifiers) than AMABs, and AFABs more often fell into cluster 4 (female identifiers) than AMABs (see Table 4). Thus, while ASAB did not have a significant main effect, nor an interaction effect, on social well-being, ASAB is related to cluster membership, which is in turn related to social well-being.

**Table 4:** Percentages of people assigned male or female at birth in each cluster, Study 2

Gender assigned at birth	Cluster 1 (male identifiers)	Cluster 2 (low identifiers)	Cluster 3 (dual identifiers)	Cluster 4 (female identifiers)
Male	48.35%	22.73%	25.21%	3.72%
Female	2.08%	17.08%	35.00%	45.83%

## Discussion

We succeeded in our main goals for Study 2: (1) To replicate the finding that a binary model is not the best fit for GI and report percentages of participants in each cluster and (2) To test whether gender non-conforming participants report lower feelings of social well-being. While in Study 1 we had an overrepresentation of female participants, in Study 2 we recruited equal numbers of AMAB and AFAB participants. We therefore reported the cluster centers and percentages of participants in each of our four clusters. As suspected, we found a larger percentage of gender non-conforming individuals (approx. 50%) than previous work (3–5%; Kuyper & Wijsen, 2014; Van Caenegem et al., 2015), suggesting that gender non-conformity is more common than previously thought. Whereas in Study 1 we found that a three-cluster model was the best fit, in Study 2 we found that a four-cluster model was the best fit. A four-cluster model is in line with previous research done in children (Martin et al., 2017) and indeed in Study 2 we find the same four clusters as previous research. It seems that in Study 1, perhaps due to our sample not having an equal sex distribution, we did not find enough participants who are low identifiers of both binary genders (cluster 2 in Study 2) as that was the missing cluster.

In Study 2, we also found that participants’ GI was related to their feelings of social well-being, namely authenticity, belongingness, self-esteem, life satisfaction and positive affect, but not directly to their self-assigned gender category or assigned sex at birth (ASAB). Specifically, participants in cluster 2 (low identifiers), who are gender non-conforming, consistently reported the lowest social well-being on all measures, and also lower well-being than the other gender non-conforming cluster (3, dual identifiers). This is in line with previous findings of Martin et al. (2017) and Pauletti et al. (2017), who found that low identifiers experienced negative consequences to social adjustment. There is also extensive literature suggesting that lack of group identifications can have negative implications on health and well-being (Jetten et al., 2012), which may provide an explanation for this finding. Additionally, participants in cluster 3 (dual identifiers) reported being less able to be their authentic selves, an important part of social inclusion (Jansen et al., 2014), than participants in cluster 1 (male identifiers). However, there were no significant differences between participants in cluster 3 (dual identifiers) and participants in cluster 4 (female identifiers) on any of the well-being measures. This suggests that being a dual identifier is not related to additional negative consequences to social well-being, compared with being a female identifier. This may be because dual identifiers are able to socially adjust quite well, in a similar way as psychologically androgynous people (Bem & Lewis, 1975) have been found to be well-adjusted because of their sex role adaptability. Moreover, our results suggest that being a male identifier is related to the highest social well-being, as compared to all other gender identifications. Interestingly, while there were no significant main or interaction effects of ASAB and self-assigned gender category on social well-being, we did find that ASAB was related to cluster membership (see below), suggesting an indirect effect on social well-being.

Lastly, we found that the distribution of people with different ASABs was different for each GI cluster membership. More AMABs were male identifiers than AFABs and more AFABs were female identifiers than AMABs. This seems to show that being brought up as a girl or boy increases identification with women and men respectively. Furthermore, we found that more AFABs than AMABs were gender non-conforming (specifically, dual identifiers) suggesting that high identification with both binary genders is more common among people who are considered women. This could be because women are the lower status group, which may lead them to want to identify more with men. In comparison, men may be motivated not to identify with women because they are the lower status group (e.g., Blau & Kahn, 2007), but we can only speculate about this.

A limitation of Studies 1 and 2 was that, while being less restrictive in that people could indicate their GI with more than one binary gender, we had only included items about the two binary genders: “man” and “woman.” As such, a main aim of Study 3 was to test how participants would respond to a multiple-identification questionnaire that included a third gender, which we investigated in Study 3. Furthermore, in Studies 1 and 2, we had used the same questionnaire for measuring cognitive GI, which we had not yet compared to any other measure of self-categorization. Our scalar measure relies heavily on participants’ understanding of the English language, and there may be a concern that variance in our data is related to participants’ different understandings of the items in the scale. As such, in Study 3, another main aim was to additionally include a second (pictorial) measure of self-categorization (as has also been done by Martin et al., 2017) to cross-validate the results of the scalar and pictorial measures, and to act as a conceptual replication of our results.

## Study 3

### Method

#### Participants

Based on a power analysis (effect size *f* = 0.20, smallest effect of GI on well-being from Study 2) we recruited 280 participants (140 AFAB, 140 AMAB; 140 identified as a man, 136 as a woman, 5 as a person with a third gender). Participants’ age ranged from 18 to 63 (*M* = 36.97, *SD* = 8.58). Participants lived in 25 different countries, with the majority living in the UK (20.28%) or Portugal (18.51%). For information about participant country of residence, education, employment, and special needs, see Supporting Information.

#### Materials

All items were measured on a 7-point Likert scale unless stated otherwise.

##### Gender Identification

We used the same GI items as in Study 2. However, in addition to administering items about men and items about women, we also administered items about a third gender (e.g. “I see myself as someone belonging to a third gender”). We chose the term *third gender*, rather than a term like *non-binary*, because it encompasses all gender identities other than “man” and “woman,” while not being too negatively loaded by sensationalist media (items about men α = .94, items about women α = .95, items about third gender α = .92; see Appendix 1).

##### Overlap of Self and Group

As a pictorial measure of self-categorization, we administered three items (one about men, one about women, one about a third gender) asking participants to indicate which image, out of seven, best represents who they are in relation to each group. The images were Venn diagrams with one circle representing “you” and the other representing a gender group, which went from being very far apart to overlapping entirely (Schubert & Otten, 2002).

##### Social Well-Being

We administered the same Inclusion Scale (authenticity α = .96, belongingness α = .93), Self-esteem Scale (α = .89) and Life Satisfaction Scale (α = .86), as in Study 2.^9^

#### Procedure

Participants were recruited through Prolific, using restrictions to recruit equal numbers of people in terms of ASAB. They were redirected from Prolific to the survey’s Qualtrics link and asked to answer all questions. First, they were shown a text explaining that people can feel like a gender besides man or woman, that for the purposes of this experiment we will call any other gender besides man or woman a “third gender,” and assuring them that any feelings they may have regarding their gender were perfectly normal. Next they filled in the scales in the following order: GI scale, Overlap of Self and Group items, Inclusion Scale, Self-Esteem Scale and Life Satisfaction Scale, Precarious Manhood and Womanhood (the latter is beyond the scope of this paper, but can be found in the Supporting Information). Items within scales were administered in a random order. Lastly, they answered demographic questions, read a debriefing, and had a chance to retract their data from further analysis. Participants received payment for their participation.

## Results

### Gender Identification

As in Studies 2 and 3, we calculated GIW and GIM scores based on the means of the GI items about women and items about men, respectively. Additionally, we calculated GIT (gender identification with a third gender) scores based on the means of the cognitive GI items about a third gender group. For means, standard deviations and correlations of GI, see Table 5. The correlation between GIM and GIW was much higher in this sample than in Studies 1 and 2. This suggests that adding a third category to the scale alters people’s responses to the items about binary categories. Furthermore, while our sample identified about equally with men and women (at around scale midpoint), *t*(280) = 1.13, *p* = .26, the overall identification with a third gender was significantly lower than both GIM, *t*(280) = 16.40, *p* < .001, and GIW, *t*(280) = 17.67, *p* < .001.

**Table 5:** Descriptive statistics and correlations of gender identification with women, gender identification with men and gender identification with a third gender, Study 3

	GIW	GIM	GIT
Descriptive statistics	*M* = 4.33 *SD* = 2.01	*M* = 4.07 *SD* = 1.95	*M* = 1.90 *SD* = 1.13
Correlation with GIW		*r*(278) = − .90* *p* < .001	*r*(278) = .01 *p* = .86
Correlation with GIM			*r*(278) = .05 *p* = .45

Absolute range of GIM, GIW, and GIT: 1–7

Several two-step cluster analyses with three inputs (GIW, GIM and GIT) revealed that a 4-cluster model (as compared to 2, 3, 5 and 6-cluster models) was the best fit in terms of percentage of variance, Bayesian Information Criterion (*BIC* = 298.48) and silhouette of cohesion (silhouette ≥ .5) and separation combined. The cluster membership of each participant was saved to perform further analyses. The clusters can be interpreted as: (1) Male identifiers, (2) Multiple identifiers with male tendencies, (3) Multiple identifiers with female tendencies and (4) Female identifiers (see Table 6 for cluster centers and percentages of the sample in each cluster). This model differs from that of Study 2, in that there is no cluster that identifies lowly with both men and women. Again, it seems that adding a third gender category altered the way participants thought about the items about binary categories. For the purposes of replication, we also ran a cluster analysis with two inputs (GIM and GIW), as well as a cluster analysis with the OSG measure, and found that a three-cluster model was the best fit for our data. This shows that a binary model was not the best fit, even when only including GIM and GIW as inputs. The results of this are beyond the scope of this paper, but can be found in the Supporting Information.

**Table 6:** Cluster centers for gender identification with women, gender identification with men, and gender identification with a third gender, and percentage of participants who fell into each cluster in Study 3

	Cluster 1 (male identifiers)	Cluster 2 (multiple identifiers with male tendencies)	Cluster 3 (multiple identifiers with female tendencies)	Cluster 4 (female identifiers)
Cluster center for GIW	2.27	3.41	5.82	6.35
Cluster center for GIM	5.97	5.00	3.14	1.97
Cluster center for GIT	1.33	3.64	2.92	1.24
Percentage of overall sample	35.59%	17.08%	13.17%	34.16%

Absolute range of GIM, GIW, and GIT: 1–7

Once again, we investigated whether gender cluster membership differed depending on country of residence, as one might expect gender and culture to interact. As in Study 2, we found a non-significant result in a chi square testing this relationship (*χ*^2^ = 82.56, *p* = .19).

### Overlap of Self and Group

To investigate whether the GI scale used in Studies 1 and 2 measured self-categorization in a similar way that other measures do, we compared our GI means with the Overlap of Self and Group measure. For means, standard deviations and correlations of the Overlap measure, see Table 7. We performed a Pearson’s correlation analysis to compare GIM, GIW and GIT with each corresponding Overlap question, respectively. We found a significant and medium-large correlation between GIW and the Overlap of Self with Women measure (*r*(278) = 0.77, *p* < .001), between GIM and the Overlap of Self with Men measure (*r*(278) = 0.81, *p* < .001), and GIT and the Overlap of Self with Third gender measure (*r*(278) = 0.71, *p* < .001). All correlations were positive, medium-large (*r* < 0.7) and significant. We also looked at the mean Overlap measure per cluster membership and found that the patterns corresponded to those seen in the cluster centers for the GI measure (see Table 8). This suggests that while not measuring the exact same construct, the two measures overlap greatly.

**Table 7:** Descriptive statistics and correlations of the Overlap of Self with Women, the Overlap of Self with Men, and the Overlap of Self with Third gender, Study 3

	Overlap of Self with Women	Overlap of Self with Men	Overlap of Self with Third gender
Descriptive statistics	*M* = 4.38 *SD* = 2.09	*M* = 4.13 *SD* = 2.14	*M* = 1.83 *SD* = 1.17
Correlation with Overlap of Self with Women		*r*(278) = − .64* *p* < .001	*r*(278) = .08 *p* = .19
Correlation with Overlap of Self with Men			*r*(278) = .22 *p* = .71

Absolute range of each Overlap measure: 1–7

**Table 8:** Descriptive statistics for Overlap of Self and Group per cluster, Study 3, M(SD)

Measure	Cluster 1 (male identifiers)	Cluster 2 (multiple identifiers with male tendencies)	Cluster 3 (multiple identifiers with female tendencies)	Cluster 4 (female identifiers)
Overlap of Self with Women	2.68 (1.38)	3.72 (1.70)	5.19 (1.43)	6.17 (1.35)
Overlap of Self with Men	5.96 (1.37)	4.81 (1.91)	3.38 (1.34)	2.17 (1.09)
Overlap of Self with Third gender	1.36 (0.76)	2.83 (1.58)	2.84 (1.01)	1.43 (0.72)

Absolute range of each Overlap measure: 1–7

### Effect of Gender Identification on Social Well-Being

To investigate whether we can replicate the findings of Studies 1 and 2, that GI cluster membership is related to a person’s social well-being, we ran a MANOVA with GI cluster membership (4 levels) as IV and the following DVs: mean feelings of authenticity and belongingness in society, self-esteem and life satisfaction. We found a main effect of GI cluster membership on all measures of social well-being; belongingness, *F*(3, 277) = 6.92, *p* < .01, η_**p**_^2^ = 0.07; authenticity, *F*(3, 277) = 7.72, *p* < .01, η_**p**_^2^ = 0.08; self-esteem, *F*(3, 277) = 4.39, *p* < .01, η_**p**_^2^ = 0.05; except for life satisfaction, *p* = .056. We once again explored the effects of ASAB and self-assigned gender category on social well-being (using a MANOVA), and whether these variables are moderators for the relationship between GI and social well-being. Unlike in Study 2, we found a significant main effect of ASAB on life satisfaction, *F*(1, 267) = 7.37, *p* = .04, η_**p**_^2^ = 0.01, such that AMABs reported higher life satisfaction than AFABs, *M* = 4.30, *SD* = 1.35, *M* = 4.21, *SD* = 1.27, respectively. However, replicating Study 2, we found no other main or interaction effects. To investigate which clusters differed from each other in terms of the different measures of social well-being, which we had found a significant main effect of cluster membership for, we ran Tukey HSD tests, which report separately for each measure, below.

#### Authenticity

**Fig. 8:**
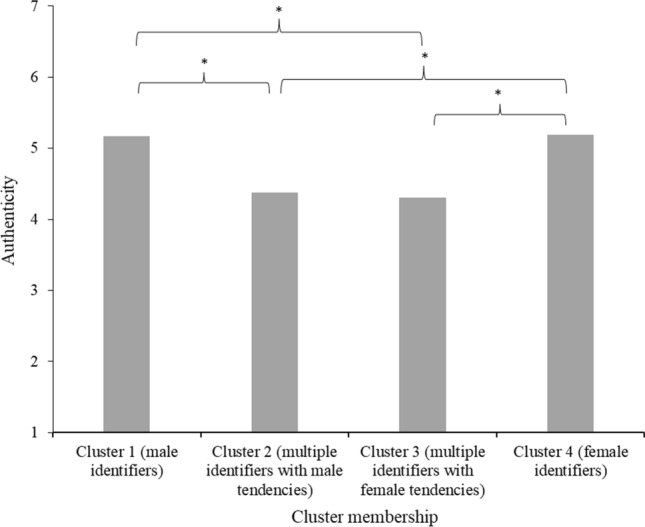
Mean feelings of authenticity in society per gender identification cluster in Study 3 *Note*. Stars (*) denote significant differences between clusters (*p* < 0.05)

The Tukey HSD revealed that people in clusters 2 (*M* = 4.38, *SD* = 1.49) and 3 (*M* = 4.31, *SD* = 1.38; gender non-conforming clusters) reported significantly lower feelings of authenticity (*p*s ≤ .005) than people in clusters 1 (*M* = 5.17, *SD* = 1.29) and 4 (*M* = 5.19, *SD* = 1.28; binary clusters) but did not significantly differ from each other. Clusters 1 and 4 also did not significantly differ from each other in terms of authenticity (see Fig. 8).

#### Belongingness

**Fig. 9:**
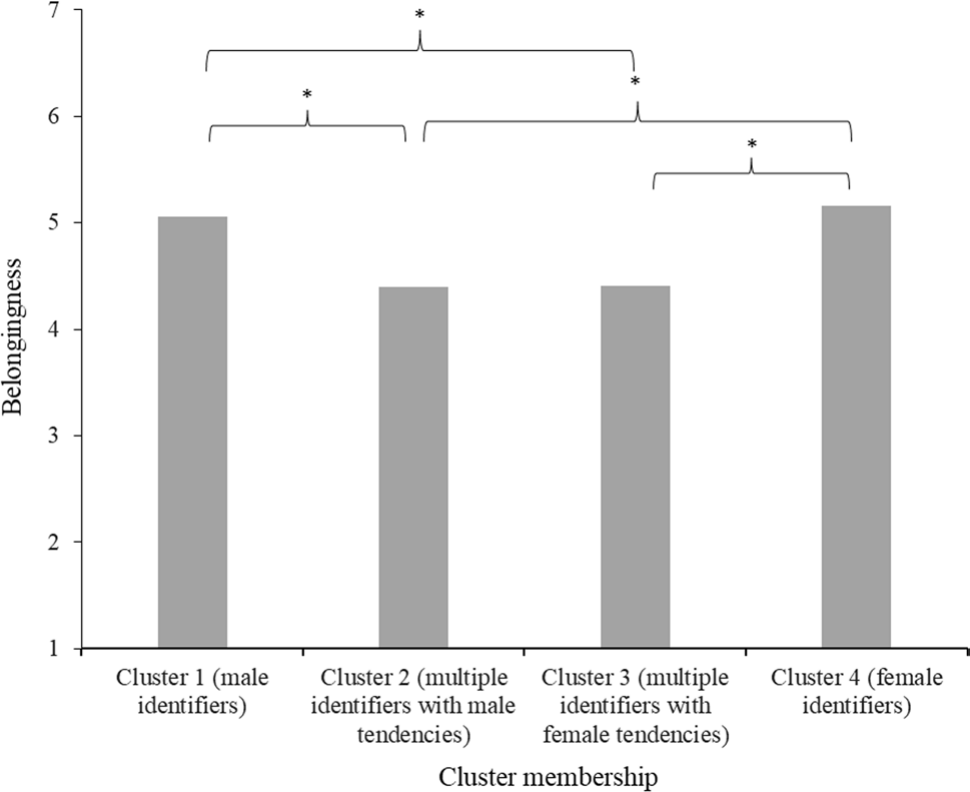
Mean feelings of belongingness in society per gender identification cluster in Study 3 *Note*. Stars (*) denote significant differences between clusters (*p* < 0.05)

The Tukey HSD revealed that people in clusters 2 (*M* = 4.40, *SD* = 1.26) and 3 (*M* = 4.41, *SD* = 1.29; gender non-conforming clusters) reported significantly lower feelings of belonging (*p*s ≤ .026) than people in clusters 1 (*M* = 5.06, *SD* = 1.15) and 4 (*M* = 5.16, *SD* = 1.19; binary clusters) but did not significantly differ from each other. Clusters 1 and 4 also did not significantly differ from each other in terms of belonging (see Fig. 9).

#### Self-Esteem

**Fig. 10:**
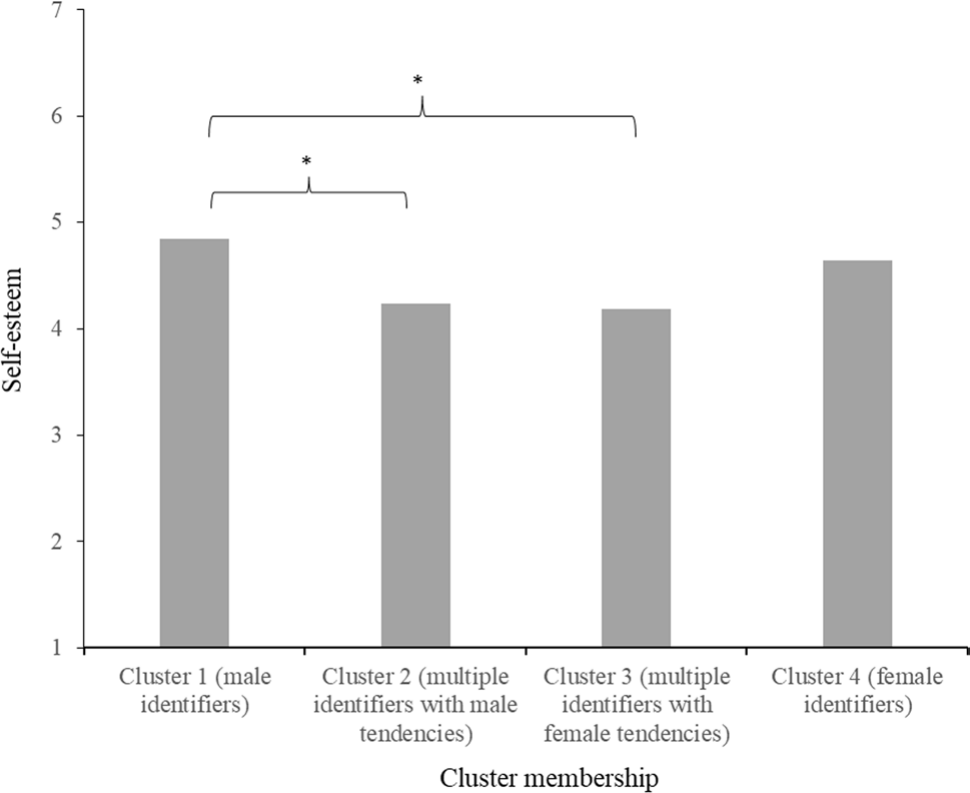
Mean feelings of self-esteem per gender identification cluster in Study 3 *Note*. Stars (*) denote significant differences between clusters (*p* < 0.05)

The Tukey HSD post-hoc test revealed that people in cluster 1 (male identifiers; *M* = 4.84, *SD* = 1.08) reported significantly higher self-esteem (*p*s ≤ .022) than people in clusters 2 (*M* = 4.24, *SD* = 1.26) and 3 (*M* = 4.18, *SD* = 1.28; gender non-conforming clusters), but not people in cluster 4 (*M* = 4.64, *SD* = 1.24). No other differences were significant, but the (non-significant) pattern of results follows that of the other variables of social well-being (see Fig. 10).

To see whether our measures of social well-being were related to one another, we ran a Pearson’s correlation and found that all above measures, as well as life satisfaction, were significantly correlated with one another (see Table 9).

**Table 9:** Correlations between social well-being measures in Study 3

Measure	Authenticity	Belongingness	Self-Esteem
Belongingness	*r*(278) = .83 *p* < .001		
Self-Esteem	*r*(278) = .41 *p* < .001	*r*(278) = .47 *p* < .001	
Life Satisfaction	*r*(278) = .46 *p* < .001	*r*(278) = .51 *p* < .001	*r*(278) = .68 *p* < .001

### Differences in Gender Identification Depending on Assigned Sex at Birth

To explore the relationship between ASAB and GI cluster membership, we ran a chi-square test with ASAB as IV and cluster membership as DV. It revealed a significant difference in cluster memberships (*χ*^*2*^ = 218.60, *p* < .001) across AMABs and AFABs (see Table 10). Post-hoc *z*-scores revealed that the proportions of AFAB and AMAB participants in all clusters differed significantly (*p* ≤ .05), with more AMABs falling into clusters 1 (male identifiers) and 2 (multiple identifiers with male tendencies), and more AFABs falling into clusters 3 (multiple identifiers with female tendencies) and 4 (female identifiers).

**Table 10:** Percentages of people assigned male or female at birth in each cluster, Study 3

Assigned sex at birth	Cluster 1 (male identifiers)	Cluster 2 (multiple identifiers, male tendencies)	Cluster 3 (multiple identifiers, female tendencies)	Cluster 4 (female identifiers)
Male	7.50%	25.90%	2.16%	1.44%
Female	1.43%	7.86%	24.29%	66.43%

## Discussion

We succeeded in our main goals for Study 3: (1) To investigate the effect of adding a third gender to our multiple-identification model, and (2) To cross-validate our GI scale with a pictorial measure of Overlap of Group and Self, both of which are thought to measure self-categorization as a social group member (Turner & Reynolds, 2011).

Adding a third gender category to our multiple-identification model in Study 3 affected the results in that our correlation between GIM and GIW was noticeably higher, while still negative, than in Studies 1 and 2. Furthermore, we did not find any low identifiers of binary genders in our model in Study 3, as opposed to Study 2. It seems that the addition of a third gender category may deter participants from picking the lower scale points for binary genders. Self-categorization theory (Turner & Reynolds, 2011) poses that we categorize ourselves as a member of an in-group, as compared to an out-group. Redefining the gender groups that participants were answering questions about (going from two to three), could therefore have caused them to rethink which groups are an in-group and which an out-group. In this case, the clearer out-group might have been people with a third gender, which would increase self-reported identification with the in-group, namely binary genders. Importantly, we did find two clusters of people who reported medium identification with a third gender; the percentages of people falling into those clusters were sizeable (30.25%). This suggests that a sizeable amount of the sample understood a feeling of having a third gender outside of the binary, at least to some extent, and were not “put off” by answering questions about this. Considering our sample had a majority of cisgender, heterosexual participants, this is a notable and novel finding. Only five people in our sample chose “person with a third gender” as their explicit self-assigned gender category, while many more self-categorized to a certain extent as a third gender, showing the difference in results when measuring gender on a scale rather than as a categorical variable.

We found that our GI measure, which is comprised of a mean score of several items rated on a 7-point Likert scale, correlated highly and positively with the Overlap measure, which uses a single pictorial item to assess how much overlap a person perceives between their own identity and that of a certain group. Furthermore, participants’ cluster centers for the scalar GI measure were comparable to the mean Overlap score for each cluster. All in all, this suggests that the two measures capture similar constructs, and we would come to similar conclusions using either measure.

Furthermore, replicating Study 2, we investigated how participants in different gender identification clusters differed in terms of social well-being. We found that participants in cluster 2 and 3 (both gender non-conforming clusters) reported lower feelings of authenticity and belongingness, than participants in clusters 1 and 4 (both binary clusters). Furthermore, we found that participants in the gender non-conforming clusters reported having lower self-esteem than male identifiers (cluster 1), but did not differ from female identifiers (cluster 4). With regards to authenticity, this finding is in line with findings from Study 2, where we found that participants in gender non-conforming clusters reported lower authenticity than participants in binary clusters. For belongingness, our finding in Study 3 differs somewhat from that in Study 2, where we only found that participants in one of the gender non-conforming clusters reported lower self-esteem than all other clusters. We think this is to do with the difference in content of each cluster in Study 3, as compared to Study 2. As discussed, in Study 3 we found that participants generally did not report identifying lowly with both binary genders, since providing a third gender option seemed to affect participants’ reported binary gender identification levels. In Study 2, the group with the lowest reported social well-being (including belongingness) were participants who identified relatively low with both binary genders (but for whom we did not know how much they identified with a third gender). Lastly, our findings regarding self-esteem in Study 3 are somewhat in line with our findings in Study 2. Specifically, in Study 2 we had found that male identifiers reported having higher self-esteem than female identifiers and participants in one of the gender non-conforming clusters (low identifiers). In Study 3 we thus replicate that both gender non-conforming and female identifiers have lower self-esteem than male identifiers.

Finally, replicating Study 2, we explored the distributions of AFAB and AMAB participants in each gender identification cluster. Our findings were in line with what one might expect, namely that more AFAB participants were in clusters that identified relatively highly with women, while more ASAB participants were in clusters that identified relatively highly with men.

## General Discussion

Across three empirical studies, we shed new light on gender non-conformity and its relationship with social well-being. The main aim across all studies was to investigate the prevalence of adult gender non-conformity in a novel way: using social identification questionnaires and a cluster analysis inspired by Martin et al. (2017). We found the same result across all studies: that a binary model of gender is not as good a fit for our data as a multiple-identification model of gender. Using our multiple-identification models, we found a larger percentage of gender non-conformity in the general population than some of the previous literature (Kuyper & Wijsen, 2014; Van Caenegem et al., 2015). This suggests that gender non-conformity is a more common occurrence than previously thought. We argue that the questionnaires we provided participants gave them more space to express how they feel about their gender than in previous studies (e.g., Joel et al., 2014). This was due to the number of items used being higher and more diverse in terms of facets of gender that were addressed.

### Gender Non-Conformity in Each of Our Studies

A discrepancy between results in the studies are the *number* of clusters found in the multiple-identification model: three in Study 1, four in Studies 2 and 3. We argue that because the sample in Studies 2 and 3 were more representative of the general world population in terms of ASAB (approx. 50/50), results from Studies 2 and 3 are likely closer to the true amount of gender non-conformity in the general population.

We also found differences in the content of the clusters between studies. Notably, in Study 2 we found a cluster of people who identified lowly with both binary genders, while no such cluster was found in Study 3. This was likely because of a difference in design: in Study 3 we measured GI with three gender groups, rather than two, which we had not done in previous studies. This difference in results between Studies 2 and 3 can be explained using self-categorization theory (Turner & Reynolds, 2011). Self-categorization theory states that intergroup identification (which we measured) is the amount to which one categorizes with one group as compared to another group. The comparison of groups therefore makes a difference in self-reported identification. Comparing men, women, and people with a third gender, may make the comparison between binary and non-binary gender groups more salient than just comparing men with women.

Moreover, while one might expect a difference in gender identification between people of different countries because gender is culturally constructed (Newman, 1995), our results do not suggest that this is the case. In Studies 2 and 3 we found non-significant chi square results for the relationship between gender cluster membership and country of residence. We refrained from performing this analysis in Study 1 because of our less diverse sample, with most of our participants being from majority White, Westernized countries. In our studies, we made a start in investigating the effect of culture on gender identification, and our sample was multi-national in each of the three studies. However, the relationship between gender identification and country of residence can be investigated further, by reducing the White, Westernized bias in sampling further, and performing a more direct comparison between countries with different gender ideologies.

### How Gender Non-Conformity and Social Well-Being Are Related

Adding to Study 1, in Studies 2 and 3 we examined whether and how GI relates to social well-being. Importantly, across both studies we found that gender non-conforming people consistently reported feeling less able to be their authentic selves in society, as compared to binary identifiers, showing that our multiple-identification model has real consequences. Thus, even though across both studies we had different methods, as well as different findings regarding gender identification clusters, we found that authenticity seemed to play a big role for gender non-conforming participants in both studies. A meta-analysis by Sutton (2020) showed that authenticity is consistently positively related to important well-being outcomes, thus further highlighting the need for people to be able to express their authentic selves. This is an important indicator that acceptance of gender non-conformity in society needs to be addressed, so that they feel more able to be authentic.

Furthermore, there were some social well-being findings that were specific to each study. In Study 2, we found that low identifiers of gender (cluster 2) reported significantly lower well-being (all measures) than participants in all other clusters, including dual identifiers (cluster 3, i.e., the other gender non-conforming cluster). Identification with any group tends to be related to increased psychological well-being (e.g., Brook et al., 2008), so reporting no or low identification with all groups in a survey would likely cause participants to report lower well-being than those who do identify with (a) group(s). On the other hand, dual identifiers (cluster 3) in Study 2 seemed to be relatively well-adjusted, sometimes even better adjusted than female identifiers (cluster 4), in terms of social well-being (except authenticity). This finding is in line with previous research in children (Martin et al., 2017) and adults (Andrews et al., 2019; Endendijk et al., 2019) who found negative effects of low identifications and positive effects of dual identifications on various measures of social well-being (e.g., self-esteem, efficacy). This finding is also in line with research from Bem and Lewis (1975), who found that psychologically androgynous people, who report similar (relatively high) amounts of masculine and feminine traits, tend to be more adaptable and therefore have higher feelings of well-being. However, it should be noted that gender identification is a different concept from gender roles (measured through personality traits), as also pointed out by Martin et al. (2017) and Pauletti et al. (2017). One can be psychologically androgynous but identify highly with one binary gender (for more information about how our measure of gender identification is related to scores on the BSRI, see Supporting Information). Being adaptable in terms of personality traits may be beneficial but feeling excluded by a society that does not accept feelings about gender beyond the binary may take its toll on people’s well-being. In line with this, in Study 3, we found that participants in both gender non-conforming clusters reported lower feelings of belongingness in society than binary identifiers, as well as lower self-esteem than binary male identifiers. Therefore, even though gender non-conforming participants in Study 3 were relatively high identifiers of several genders, they reported suffering on several measures of social well-being. This further highlights the potential differences between psychological androgyny and gender non-conformity in terms of identification.

In both Studies 2 and 3, we also explored how ASAB and self-assigned gender category were related to social well-being, and how they affected the relationship between GI and social well-being. We theorized that ASAB and self-assigned gender category are indicators of how one is perceived by others, and that this could influence social well-being. For instance, someone who is assigned male at birth is typically raised as a boy and is often perceived by others as a boy or man throughout his life. Men tend to be afforded more privileges than women (Eagly, 1983) and therefore may feel more included by society in general. In Study 2, we found no significant results of ASAB or self-assigned gender category (neither main nor interaction effects) on social well-being. In Study 3, we largely replicated these results, with one exception. In Study 3, we found a significant main effect of ASAB on life satisfaction, such that AMABs reported higher life satisfaction than AFABs. This is in line with the idea that people who are mostly perceived as men may have more privilege than people who are mostly perceived as women, and therefore may feel more satisfied in their lives. What remains unclear, however, is why we found this result in Study 3, but not Study 2. What was consistent across both studies though, was that the effect of gender identification on social well-being is not moderated by ASAB or self-assigned gender category. We believe this underlines the theoretical implications of our multiple-identification model, and that our self-categorization measure can be related to real psychological consequences, without it necessarily acting as a proxy for other ways of measuring gender and sex.

### Limitations

As stated, there was a discrepancy in our findings regarding the different clusters of GI whereby we found a different number of clusters, and different meanings attached to each cluster, across our three studies. This is likely to do with a lack of direct methodological replication in our studies: the measures in our survey, and the demographics of our sample, differed from study to study. Furthermore, the correlation of GIW with GIM showed a vastly different coefficient across studies. While in Studies 1 and 2, the correlation coefficients were relatively small, suggesting discriminant validity between the measures of GIM and GIW (Campbell, 1960), in Study 3, we found a high correlation coefficient between these two measures. This seems to show that, when asked to consider to what extent they identify with a third gender, participant responses shift such that GIM and GIW become highly related to one another. It should be noted that, even in Study 3, where GIM and GIW were highly related to one another, the best cluster solution for our data, with two inputs (GIM and GIW), was not binary but, rather, contained three clusters. As such, while the high correlation between GIM and GIW suggested that the two measures were on the same continuum (lack of discriminant validity), measuring both allowed us to find more than two clusters of gender identification. It is important for future researchers to investigate this finding further, and to run direct methodological replications of their studies.

In order to measure GI, we used a social identity theory (Tajfel, 1974) approach taken from Ellemers et al. (1999). However, there are alternative ways to measure gender identification. Our approach could be seen as measuring the self-investment component of gender identification (i.e., “solidarity” with a group, “satisfaction” with a group, and identity “centrality”), while there is also the self-definition component of gender identification, which measures self-stereotyping and group homogeneity (Leach et al., 2008). Measuring self-definition was not part of the scope of this paper. However, it will be important in future studies to investigate how estimates of gender non-conformity differ depending on the way we measure their identification.

Next to the different ways of measuring identification with men and women, we also did not measure how our results would change if we had used different gender terminology than “man” and “woman.” Schudson et al. (2019) demonstrated that different gender words are associated with different definitions. While the words “male” and “female” tend to be associated with biological cues, such as body shape and genitals, the words “masculine” and “feminine” tend to be associated with social cues, such as clothing. Importantly, they found that the words “man” and “woman” are associated with a little bit of both types of gendered/sexed information: biological and social. Had we used words associated with just biology, for instance, we might have found that a binary model (two clusters) may have had the best statistical fit, as biological sex is seen as more fixed than gender (Skewes et al., 2018). On the other hand, had we used words associated with social information, for instance, we might have found that a three-cluster model would have been the best fit, similar to Bem (1974; “masculine,” “feminine,” and “androgynous” people).

Participants in our studies were recruited from the sample of the online platform Prolific. This sample is relatively representative of the general population (Palan & Schitter, 2018). However, the majority of participants who are active on Prolific are White and British. In Study 1, we found a much higher percentage of White participants and participants living in the UK than in any other country. This is not ideal since the changes in the gender narrative are happening world-wide and not just in the UK. However, in Study 2 we had more participant requirements and therefore our sample was much more international and ethnically diverse, meaning the White British-centric bias of Study 1 was reduced. Since results of both studies mostly converge, we assume that the UK bias in Study 1 was not detrimental to our results. Furthermore, our measurements were all explicit and self-reported. In future research we aim to use more implicit measures of gender identification, to further grasp the prevalence and implications of gender non-conformity.

Finally, while the main scope of this paper was about strict gender categorizations that are in place in society, gender may not be the only social category that is falsely assumed to be binary. Social categories such as race (Black or White) or age (old or young) might behave in similar ways as the gender results described in this paper. It is worth noting that we made a start at comparing binary gender and binary ethnic categories and found that ethnic categories did not behave the same way as gender categories; the details of this can be found in the Supporting Information.

### Practical Implications

We believe that the data reported in this paper speak to the current rise in gender-inclusive interventions (third gender markers in legal documents, e.g., Dunne & Mulder, 2018; gender-neutral toilets, e.g., Gershenson, 2010; unisex clothing, e.g., Park, 2014; gender-neutral language, e.g., Hord, 2016). These interventions are designed to include people who identify as “non-binary” (their self-assigned gender category is “non-binary”). However, we believe that these interventions could help increase feelings of inclusion in anyone who self-categorizes, in one way or another, in a gender non-conforming way. We have shown that the number of people who identify in a gender non-conforming way may be much larger than previously thought, and that gender non-conformity is related to social well-being, including how included or excluded people feel by society. We argue that an increase in gender-inclusive interventions may therefore be a positive change for many, including cisgender, heterosexual people who identify in a gender non-conforming way. However, more research is needed to draw a direct link between gender-inclusive interventions and people’s higher social well-being, as well as how to design these interventions to maximize positive effects.

### Conclusion

In this paper, we show that a binary model of gender (“man” vs. “woman”) is limited. Across three studies, we found that a binary model does not fit the general population as well as a multiple-identification model of gender. This means that measuring gender in a binary way (women are only asked how much they identify with women, and vice versa) misses some of the complexities and nuances of people’s gender identification. If we continue to do so, we are likely to draw a skewed picture in (social psychological) research. Furthermore, there are real consequences to identifying in gender non-conforming ways, as we found that individuals identifying in a gender non-conforming way often suffer from lower social well-being (particularly, feeling like they cannot be their authentic selves). Our research therefore reveals why maintaining a binary system in society may be harmful to a significant proportion of the population.

### Supplementary Information

Below is the link to the electronic supplementary material.

Supplementary file1 (DOCX 993 KB)

## Data Availability

A data package has been archived and can be viewed at https://osf.io/2s93m/?view_only=36f11c0e5654458a83c0a12dc8f340a0 (access can be requested via the link).

## Appendix 1: Three-Way Gender Identification Scale

1. I identify with women
2. I identify with men
3. I identify with a third gender
4. I identify as a woman
5. I identify as a man
6. I identify as a person with a third gender
7. I am like women
8. I am like men
9. I am like people with a third gender
10. Women are an important reflection of who I am
11. Men are an important reflection of who I am
12. People with a third gender are an important reflection of who I am
13. I see myself as someone belonging to the group of women
14. I see myself as someone belonging to the group of men
15. I see myself as someone belonging to a third gender
16. I have a lot in common with women
17. I have a lot in common with men
18. I have a lot in common with people with a third gender

## Footnotes

1 For a more detailed explanation of our power analysis reasoning, please see Supporting Information.

2 We checked for differences in cluster memberships depending on demographic profile by means of chi-square analyses and Pearson’s correlations, but found no significant results.

3 We additionally asked participants about their emotional connection to each gender group, and their evaluation of each gender group, which are other subcomponents of gender identification, in line with Ellemers et al. (1999). There were several issues with these measures, such as cross-factor loadings, low reliability, and lack of replication between Study 1 and Study 2 (which is also why we excluded these measures from Study 3). Because of this, the results of these measures are beyond the scope of this paper but can be found in the Supporting Information.

4 You can find the results of these questionnaires in the Supporting Information. The exceptions to this are the questions about feminine and masculine norms. We asked participants to fill out shortened versions of the Conformity to Feminine Norms Inventory (CFNI; Mahalik et al., 2005) and the Conformity to Masculine Norms Inventory (CMNI; Mahalik et al., 2003). The goal, initially, was to measure a general conformity to feminine or masculine norms. However, a recent study has shown that the CMNI does not reliably measure a general conformity to masculine norms and is only reliable for measuring conformity to specific norms (Hammer et al., 2018), which we were unable to do with our shortened versions of the questionnaire. We concluded that the CFNI is similarly problematic and therefore refrained from analyzing data from either measure.

5 We also ran separate analyses within male and female participants on the relationship between GIM and GIW. We found that the pattern of results of male and female participants were similar (with one result being non-significant in male participants but significant in females, likely due to lower male sample size), meaning we come to the same conclusions regardless of the participant’s gender. The details of these analyses can be found in the Supporting Information.

6 For more information about the reasoning for our power analysis, please refer to Supporting Information.

7 The word “ethnicity,” rather than “race,” was used in Prolific’s pre-screening question at the time. Prolific have since changed their pre-screening question and use the word “race” instead, which more accurately reflects the answer options they provide (e.g., White) which are racial, rather than ethnic, categories. However, to stay consistent with the wording that was given to our participants, we have used the term “ethnicity” throughout this paper.

8 We additionally asked participants about their emotional connection to each gender group, and their evaluation of each gender group, in line with Ellemers et al. (1999). Similarly as in Study 1, the results of this are beyond the scope of this paper but can be found in Supporting Information.

9 We chose not to include the Positive and Negative Affect Schedule (PANAS), as in Study 2, because we were least interested in this measure. The other measures of social well-being were more easily generalizable to social well-being more broadly, which better fit the aim of Study 3.
