# Supplementary material for: Gender Identification Beyond the Binary and Its Consequences for Social Well-Being
Source: Arch Sex Behav. 2022 Nov 14;52(3):1073–93. doi: 10.1007/s10508-022-02453-x (PMC10102103; doi:10.1007/s10508-022-02453-x)
Supplement: Supplementary file 1 — Supplementary file1 (DOCX 993 KB) [file 10508_2022_2453_MOESM1_ESM.docx]

**Supporting Information**

**Index**

**Study 1 2**

Power analysis 2

Additional information about participant demographics 2

Extra measures: Gender identification 3

Extra measures: Gendered traits 4

Extra measures: Belief in gender binary 4

Results: Gender identification factor analysis 4

Results: Emotional GI 5

Results: Gendered traits 5

Results: Relationship between GI and gendered traits 5

Results: Splitting data into males and females 6

Results: GI and beliefs about gender (binary or not) 7

**Study 2 7**

Power analysis 7

Extra information about demographics 7

Extra materials: Belief in gender binary 9

Extra materials: Opinions about legal recognition of non-binary genders 9

Extra measures: comparing gender and ethnicity 10

Results: Separate post hoc tests for relationship between GI and positive affect, for White and non-White participants 11

Results: Beliefs about gender binary and opinions about article content 11

Results: Separate post hoc tests for relationship between GI and opinions about

shown article 14

Results: other components of GI 20

Results: Differences between the social constructs of ethnicity and gender 21

**Study 3 23**

Participant demographics 23

Extra measures: Precarious Manhood and Womanhood 24

Results: two-way cluster analyses (GIM and GIW) 24

Results: relationship between GI and beliefs of precarious manhood and womanhood 26

**References 27**

**Appendices 29**

**Study 1**

**Power analysis.** Effect sizes of differences between gender identification cluster types in Martin, Andrews, England, Zosuls and Ruble’s (2017) study in children were all large (all η_p_^2^ η 2 {\displaystyle \eta ^{2}} = 0.55 - 0.97). Given that, and that the number of groups we expected to find were four (OG-S, C-GS, BG-S and LG-S, as in the paper by Martin et al., 2017) for a power of 80% we would have needed to recruit 14 people. However, we expected that the effect size of gender identification types would not be as large in adults as in children, due to many more years of experienced binary gender categorization, gender stereotypes, and generally more developed gender biases. As this dual-identification approach has never been tested in adults, we made our own estimation of the effect size. We expected that the effect size in adults would be around medium (approx. Cohen’s *f* = 0.25). This estimate was also based on a paper by Richard, Bond and Stokes-Zoota (2003) who found that the mean effect size across all social psychology experiments *is* *f* = 0.225 (to our knowledge, no meta-analysis of effect sizes of differences in gender or social identification has been done; we thus cannot base our estimation on a more specific meta-analysis). There was no reason to expect that the effect size of the present study would be vastly different from effect sizes found in social psychology in general. With this estimated effect size in mind, we needed to collect a minimum 179 participants for 80% power.

**Additional information about participant demographics.** Participants were distributed reasonably evenly across education levels, with the majority describing their level as “Some college/university” (see Table 1 for percentages of education levels in our sample). The majority of participants were employed full time or employed part time (see Table 2 for percentages of employment situations in our sample). Our sample was predominantly Caucasian (79.67%), and predominantly living in the UK (55.85%, see Table 3) as is often the case on Prolific. We also asked participants to indicate whether they considered themselves to be a feminist (on a 7-point Likert scale going from 1 = highly disagree, to 7 = highly agree, *M* = 4.18, *S.D.* = 0.16) and whether they considered themselves to be LGBT+ (on the same Likert scale, *M* = 1.92, *S.D.* = 0.14) as these factors have been found to be important to consider when researching gender identification (Burch, 1993; Henderson-King & Stewart, 1994; Kuper, Nussbaum, & Mustanski, 2012).

**Table 1**

*Percentage of participants in Study 1 per education level*

| Education level | Percentage |
| --- | --- |
| Less than high school | 1.09% |
| High school graduate | 21.98% |
| Some college/university | 35.16% |
| Bachelor degree | 29.67% |
| Master degree | 10.99% |
| Doctorate | 1.09% |

**Table 2**

*Percentage of participants in Study 1 per employment situation*

| Employment Situation | Percentage |
| --- | --- |
| Employed full time | 42.86% |
| Employed part time | 25.82% |
| Unemployed looking for work | 6.04% |
| Unemployed not looking for work | 4.95% |
| Retired | 1.10% |
| Student | 18.68% |
| Unemployed due to disability | 2.75% |

**Table 3**

*Percentage of participants in Study 1 per country of residence*

| Country of residence | Percentage |
| --- | --- |
| Australia | 1.60% |
| Canada | 1.06% |
| Czech Republic | 1.06% |
| Estonia | 0.53% |
| France | 1.60% |
| Germany | 2.13% |
| Greece | 2.66% |
| Hungary | 1.60% |
| Ireland | 0.53% |
| Israel | 0.53% |
| Italy | 5.32% |
| Mexico | 1.60% |
| Portugal | 6.38% |
| Slovenia | 1.60% |
| Spain | 2.66% |
| Sweden | 0.53% |
| Switzerland | 0.53% |
| United Kingdom | 55.85% |
| United States | 12.23% |

**Extra measures: Gender identification.** Across Studies 1 and 2, we additionally explored three different facets of social identification: cognitive identification, which is the extent to which one self-categorizes as a member of a social group; emotional identification, which indicates the emotional connection one feels with a group; and evaluative identification, which refers to the way in which one evaluates a group (Bergami & Bagozzi, 2000; Ellemers et al., 1999). Each of these is related to different aspects of group features. Cognitive identification, for instance, is related to group size, while evaluative identification is related to group status. We measure all three components of GI, but focus on cognitive GI in the paper, as it is the most fundamental aspect of social identity (e.g., Turner & Reynolds, 1987) and therefore the most relevant for the purposes of this paper.

To test these three components, we pooled items from two existing questionnaires: the Ellemers et al. (1999) social identification scale adapted to gender, and the gender identification (GI) scale from Derks, Van Laar and Ellemers (2009). Using theory and confirmatory factor analyses, each item was located into one of three scales: cognitive GI (these were the ones used in the paper), emotional GI and evaluative GI; the latter two of which are described below under a different header (see also Appendix A). Furthermore, we wanted to measure identification with two gender groups: men and women. All items described below were therefore administered twice, with the items being identical in both cases, other than the replacement of the word “men” with “women” and vice versa. They were answered on a Likert scale going from 1 (highly disagree) to 7 (highly agree).

***Emotional gender identification.*** To test the emotional commitment of participants to each gender group, six items were administered (three about men, three about women; e.g., “I [would] dislike being a woman” [reverse coded]; items about men α = .75, items about women α = .72).

***Evaluative gender identification.*** To test participants’ value connotation of each gender group, we administered six items (three about men, three about women; e.g., “I have little respect for [other] men” [reverse coded]; items about men α = .51, items about women α = .57)^3^.

**Extra measures: Gendered traits.**

***Bem’s Sex-Role Inventory (BSRI).*** To measure the extent to which participants perceived their personality traits as typically male or typically female, we administered 14 items from the BSRI (Bem, 1974). Participants were asked to rate, on a Likert scale going from 1 (not typical at all) to 7 (highly typical), how typical a specific trait is for men, how typical it is for women, and how typical it is for themselves. We chose items such that we had equal numbers (7 each) of stereotypically male (e.g., “strong”, “aggressive”) and stereotypically female (e.g., “warm”, “gullible”) items (items about men α = .83, items about women α = .78).

**Extra measures: Belief in gender binary.**

We included a question about belief in ‘gender-as-binary’ or ‘gender-as-spectrum’, which was taken from a U.S. national poll (Fusion.net, 2015). The question was “Some countries, including India, recognize a third gender that is neither male nor female. Which more closely aligns with your view?”. There were three answer options: (a) “There are only two genders, male and female”, (b) “Gender is a spectrum, and some people fall outside of binary categories” and (c) “I don’t know”.

**Results: Gender identification factor analysis.** We investigated which items of gender identification (GI) would load onto male and female factors separately in order to inform us which items to include in our subsequent dual-identification model (see Table 4). Items that do not load onto separate male and female factors are highly correlated and can be said to represent the same construct (e.g., identification with humans in general, rather than men or women). Our dual-identification model requires items to be about men and women specifically to be able to compare the two. We ran a confirmatory factor analysis (oblimin rotation) with two factors including all cognitive, emotional, and evaluative measures of GI. It revealed that 20 out of 26 items (half of which are about men, the other half about women) loaded onto male and female factors consistently. It is worth noting that we found that only the cognitive (how one self-categorizes) and the emotional (how emotionally connected one feels to a gender group) GI items loaded onto two binary gender factors. How one evaluates a gender group (evaluative GI) is thus highly positively related to how one evaluates the other gender group and not suitable for investigating dual-identifications of gender. However, it should be noted that the evaluative scale was also dismissed due to low reliability.

**Table 4**

*Factor loadings of each cognitive GI item into “male” and “female” components, Study 1*

|  | Component 1 (female) | Component 2  (male) |
| --- | --- | --- |
| “I identify with (other) women”  “I identify with (other) men” | .87 | .77 |
| “I am like (other) women” | .79 |  |
| “I am like (other) men” |  | .80 |
| “Women are an important reflection of who I am”  “Men are an important reflection of who I am”  “I see myself as someone belonging to the group of women” | .76  .79 | .67 |
| “I see myself as someone belonging to the group of men” |  | .74 |
| “I have a lot in common with (other) women” | .90 |  |
| “I have a lot in common with (other) men” |  | .84 |
| “I feel involved with (other) women” | .65 |  |
| “I feel involved with (other) men” |  | .56 |
| “I feel closely connected with (other) women” | .76 |  |
| “I feel closely connected with (other) men” |  | .67 |

*Note.* Only factor loadings > .3 are shown.

**Results: Emotional GI.** We found a significant difference in emotional GIM (GIM_e_) and emotional GIW (GIW_e_); respectively *M* = 4.29, S*.D*. = 1.54, min. = 1.00, max. = 7.00; *M* = 4.79, *S.D.* = 1.48, min*.* = 1.00, max. = 7.00; *t*(181) = 2.45, *p* = .01, Cohen’s *d* = 0.33. To test whether GIM_e_ and GIW_e_ are related, we ran a Pearson’s correlation which revealed a medium negative correlation between GIM_e_ and GIW_e_ (*r* = -.67, *p* < .01, see Figure 1). This indicates that GIW_e_ is more strongly negatively correlated with GIM_e_, than cognitive GIM and cognitive GIW are.

**Results: Gendered traits.** We investigated whether or not self-reported gender typicality (in terms of personality traits) followed a similar pattern as GI. Using our shortened version of the Bem Sex Role Inventory (BSRI; Bem, 1974), a ‘gender typicality’ score was computed for each binary gender and each participant. Each participant gave their personal opinion of how typical a trait is for men, for women and for themselves. The mean female and male ‘gender typicality’ scores were computed from how similar the score they gave themselves was to the score they gave a typical woman, or a typical man, respectively. Every participant thus ended up with a ‘female gender typicality’ score, representing how similar they perceive themselves to be to a typical woman, and a ‘male gender typicality’ score, representing how similar they perceive themselves to be to a typical man.

We found a significant difference between female gender typicality and male gender typicality; respectively *M* = 5.33, *S.D*. = 0.81, min. = 1.79, max. = 7.00; *M* = 5.69, *S.D*. = 0.62, min. = 3.21, max. = 7.00; *t*(181) = -5.11, *p* < .01, Cohen’s *d* = 0.38. So, while GI among all participants was more female than male, participants reported to feel, on average, significantly more similar to men than women.

A Pearson’s correlation revealed that male gender typicality correlated lowly and positively with female gender typicality (*r* = .17, *p* = .02). This shows a different pattern than GIM and GIW, which are negatively correlated with one another.

**Results: Relationship between GI and gendered traits.** We ran a multivariate multiple linear regression analysis to investigate whether male gender typicality and female gender typicality could be predicted by gender identification (GIM and/or GIW) and/or self-reported gender category (male or female).

Male gender typicality score (taken from BSRI) was predicted only by GIM (*F*(1,178) = 10.35, *p* < .01, *R*^2^ = .09; *B*_intercept_ = 4.88, *B*_GIM_ = 0.17) and not GIW or gender category. Female gender typicality was predicted only by GIW (*F*(1,178) = 16.15, *p* < .01, *R*^2^ = .16; *B*_intercept_ = 5.58, *B*_GIW_ = 0.15) and not GIM or gender category. However, the *R*^2^ scores are low suggesting that GI has a low predictive value of self-reported gender typicality.^6^

**Results: Splitting data into males and females.** Participants were asked to state their gender as part of the demographics. It was assumed that the majority of people answered this question with their biological sex/their gender assigned at birth. We therefore split participants into two groups: self-reported males (*N* = 60) and self-reported females (*N* = 121), excluding the one participant who had stated that their gender was “Other”. This was done (a) in order to find whether any of the effects reported above were driven by men or by women, and do not represent people as a whole, and (b) to investigate whether gender identification and gender typicality patterns differed between males and females. We first describe results of female participants, followed by results of male participants.

***Female participants.*** While we found dual identifications of gender, we were interested to see whether there was a mean difference between GIM and GIW in each gender group. For female participants, results showed a significant difference (*t*(120) = 11.62, *p* < .01, Cohen’s *d* = 1.05), indicating that women, on average, showed higher GIW (*M* = 5.00, *S.D*. = 0.96, min. = 2.20, max. = 6.90) than GIM (*M* = 3.33, *S.D*. = 0.98, min. = 1.10, max. = 5.70).

A Pearson’s correlation revealed a significant negative correlation between GIW and GIM in female participants (*r* = -.32, *p* < .01). Thus, in female participants, having a higher GIW is related to having a lower GIM. This follows the pattern found among all participants.

Female participants showed higher female gender typicality (in terms of traits; BSRI; *M* = 5.80, *S.D.* = 0.51, min. = 3.57, max. = 6.64) than male gender typicality (*M* = 5.23, *S.D.* = 0.85, min. = 1.79, max. = 7.00); *t*(120) = -7.16, *p* < .01, Cohen’s *d* = 0.68. Thus, women rated themselves as more similar to a typical woman than a typical man.

A Pearson’s correlation revealed a significant low positive correlation between female gender typicality and male gender typicality (*r* = .25, *p* < .01). Therefore, women who rated themselves as similar to a typical woman also rated themselves as similar to a typical man, which follows a similar pattern as results found among the whole sample.

***Male participants.*** Among all participants, there was a significantly higher GIW score than GIM. We thus wanted to find out whether this was due to the higher number of female participants in the sample. Male participants showed higher GIM (*M* = 4.83, *S.D.* = 1.01, min. = 2.70, max. = 7.00) than GIW (*M* = 3.33, *S.D*. = 1.05, min. = 1.40, max. = 7.00); *t*(59) = -8.97, *p* < .01, Cohen’s *d* = 1.16. This suggests that the higher GIW found among all participants was driven by the higher number of female participants in the sample. Furthermore, it shows that, while there are dual identifications of gender, male participants report a significantly higher GIM than GIW (a mean of 1.5 point difference on a 7-point Likert scale). This follows a similar, but gender-reversed, pattern as the results in females.

A Pearson’s correlation revealed no significant correlation between GIM and GIW (*r* = .20, *p* = .13). Thus, in male participants, having a higher GIM was not related to having a lower GIW, unlike in the sample as a whole.

No significant differences in male gender typicality and female gender typicality scores (from BSRI) were found, though male participants did feel marginally more similar to a typical male (*M* = 5.53, *S.D.* = 0.66, min. = 3.00, max. = 7.00) than a typical female (*M* = 5.47, *S.D*. = 0.76, min. = 3.21, max. = 7.00); *t*(59) = 0.53, *p* = .60, Cohen’s *d* = 0.07. A Pearson’s correlation revealed a marginally significant relationship between female gender typicality and male gender typicality (*r* = .25, *p* = .06). This suggests that, unlike women, men feel as similar to a typical man as a typical woman, and that there is no significant relationship between the two. However, this could be due to the lower male sample size than female sample size.

**Results: GI and beliefs about gender (binary or not)**

Participants were asked whether they believe that gender is a spectrum or whether they believe that gender is strictly binary. We found that 51.38% of participants held ‘gender-as-spectrum’ beliefs and 43.09% of participants held ‘gender-as-binary’ beliefs (the remaining 5.53% were undecided).

As elaborated upon in the General Introduction, we had two differing hypotheses regarding the relationship between GI and beliefs about gender. To test these, we ran a two-way MANOVA with belief in spectrum/binary and self-assigned gender as independent variables, and GIM and GIW as dependent variables. We found a main effect of gender belief on GIM; *F*(1, 167) = 4.17, *p* = .04, η**_p_**^2^ = 0.02, *M*_binary_ = 4.02, *S.D.*_binary_ = 1.33, *M*_spectrum_ = 3.34, *S.D.*_spectrum_ = 1.15; but not on GIW; *F*(1, 167) = 0.04, *p* = .84; and no interaction effect between gender belief and gender on GIM; *F*(1, 167) = 1.83, *p* = .18; and GIW; *F*(1, 167) = 0.51, *p* > .48. This suggests that a higher GIM is related to the belief that gender is binary, while a lower GIM is related to the belief that gender is a spectrum, regardless of GIW or self-assigned gender. This partially confirms the hypothesis that GI and beliefs about gender are related.

**Study 2**

**Power analysis.** Given that in Study 1 we found small effect sizes for the relationship between female and male GI, that effect sizes for relationships between authenticity and well-being are also small to medium (the smallest effect size being R^2^ = .05, from Goldman & Kernis, 2002), and that in general effect sizes in social psychology are medium (approx. *f* = 0.23, from Richard et al., 2003), we assumed that the effect sizes of our planned analyses would be medium-small (approx. *f* = 0.15). We expected to find 4 clusters of gender identity and planned to perform ANOVAs to see whether cluster membership differed between AMABs and AFABs and whether cluster membership affected six different measures of well-being. We found that we would need to recruit approx. 450 participants to achieve 80% power*.*

**Extra information about participant demographics.** The mean age of participants was 30.98 years (*S.D.* = 9.94). For ethnicity, education, employment, special needs and country of residence percentages, see Tables 5, 6, 7, 8 and 9 respectively.

**Table 5**

*Percentage of participants in Study 2 per ethnicity (from Prolific)*

| Ethnicity | Percentage |
| --- | --- |
| Asian | 22.65% |
| Black | 8.45% |
| Mixed | 12.84% |
| Other | 6.08% |
| White | 50.00% |

*Note.* Due to some data loss, the percentages may not add up to 100%.

**Table 6**

*Percentage of participants in Study 2 per education level*

| Education level | Percentage |
| --- | --- |
| Less than high school | 0.62% |
| High school graduate | 14.73% |
| Some college/university | 31.54% |
| Bachelor degree | 35.06% |
| Master degree | 15.77% |
| Doctorate | 2.28% |

**Table 7**

*Percentage of participants in Study 2 per employment situation*

| Employment Situation | Percentage |
| --- | --- |
| Employed full time | 45.44% |
| Employed part time | 19.71% |
| Unemployed looking for work | 9.75% |
| Unemployed not looking for work | 3.53% |
| Retired | 1.45% |
| Student | 18.46% |
| Unemployed due to disability | 1.67% |

**Table 8**

*Percentage of participants in Study 2 per special needs situation*

| Disability | Percentage |
| --- | --- |
| Physical | 2.28% |
| Mental | 4.56% |
| Both | 0.41% |
| Neither | 92.53% |

**Table 9**

*Percentage of participants in Study 2 per country of residence*

| Country of residence | Percentage |
| --- | --- |
| Australia | 1.87% |
| Canada | 4.36% |
| Chile | .41% |
| Czech Republic | .62% |
| Denmark | .83% |
| Estonia | .21% |
| Finland | .41% |
| Germany | 2.49% |
| Great Britain | 38.38% |
| Greece | 1.04% |
| Hungary | .83% |
| Iceland | .21% |
| Israel and the Occupied Territories | .62% |
| Italy | 6.43% |
| Latvia | .41% |
| Malaysia | .21% |
| Malta | .21% |
| Mexico | 1.66% |
| Netherlands | 1.04% |
| New Zealand | .21% |
| Nigeria | .21% |
| Korea | .21% |
| Philippines | .21% |
| Poland | 1.87% |
| Portugal | 5.83% |
| Slovenia | .41% |
| Spain | 4.36% |
| Swaziland | .21% |
| Sweden | .41% |
| Thailand | .21% |
| Turkey | .21% |
| United States of America (USA) | 19.71% |
| Virgin Islands (UK) | 3.11% |
| Zimbabwe | .21% |
| Ireland | .21% |
| Taiwan | .21% |

**Extra materials: Belief in gender binary.** We administered the same question about belief in gender binary as in Study 1 (Fusion.net, 2015).

**Extra materials: Opinions about legal recognition of non-binary genders.** We selected a recent article about the inclusion of non-binary people in society, to show to participants and investigate their feelings about the issue. The article was from BBC News about an event in the UK, but relatable to people from many countries where similar events are occurring. Since a number of different (states within) nations had recently been debating a third gender option in passports or birth, we chose a recent news story that reported an event of this type. The headline read “Campaigner for gender-neutral passports wins court challenge” (see Appendix B for full article shown to participants).

***Defensiveness.*** Participants answered three items on a Likert scale ranging from 1 (highly disagree) to 7 (highly agree; all subsequent scales were answered on the same scale), to test how defensive they felt towards the issue described in the article (adapted from Howell & Ratliff, 2014; e.g., “The issue of inclusion of non-binary individuals described in the article is irrelevant to current society”; α = .80)

***Acceptance.*** To measure how accepting participants were of the issue we administered four items (adapted from Howell, Redford, Pogge, & Ratliff, 2017; e.g., “The issue described in the article, regarding an initiative to foster inclusion of non-binary genders into our societal system, represents my true values”; α = .90)

***Positive and negative opinions.*** We wrote 11 items to test whether participants found the message of the article, and therefore the legal recognition of non-binary genders, to be positive (e.g., “I think it is good that action is taken within societies to be more inclusive of non-binary genders”; three items, α = .85) or negative (e.g., “Non-binary people are complainers and there are other problems societies should focus on”; eight items; α = .88). See Appendix C for a list of positive and negative opinion items in response to the article.

***Positive And Negative Affect Schedule (PANAS).*** This scale consists of 20 emotion words, 10 of which are positively valanced (e.g., “inspired”) and 10 are negative (e.g., “ashamed”; Watson, Clark, & Tellegen, 1988). We asked participants to reflect on how they felt while reading the article, and to rate how much they felt a certain emotion from the PANAS during that time (positive items α = .88, negative items α = .92).

**Extra measures: comparing gender and ethnicity.** Lastly, we explored the extent to which people think being gender ambivalent is not acceptable, or negative. To do so, we compared people’s opinions about dual identifications of gender with their opinions about dual ethnicities, because dual ethnicities are more common than dual genders but also stigmatized (Wilton, Sanchez, & Garcia, 2013). We explored the extent to which people feel the need to belong to their assigned binary gender group, which we also compared to their need to belong to their ethnic group in order to gain perspective. As a whole, because society is highly binary in terms of gender, participants (of any gender identification) may have a high need to belong to their assigned gender group and be motivated to increase gender group belongingness, in order not to be perceived as deviants (Schneider, 2012). We investigated to what extent people feel negatively about gender ambivalence, and feel the need to belong to their assigned binary category, because this can provide insight into the restrictions that individuals experience due to the gender binary.

***Need to belong to gender and ethnic groups.*** We wanted to measure people’s need for inclusion in their in-group, specifically their gender (assigned at birth) and their ethnic groups. To do this, we adapted the eight item scale from Leary, Kelly, Cottrell and Schreindorfer (2013) and administered it twice to all participants: once for their gender group and once for their ethnic group. Gender items included “It does not bother me when other men/women do not like me” (reverse coded) and “I try hard to avoid doing things which could lead other men/women to exclude me”. Participants who had indicated their gender assigned at birth to be male were administered all items with the word “men” (α = .77), whereas participants assigned female at birth were asked to respond to the items with the word “woman” (α = .81). Additionally, all participants had been asked to fill in their ethnicity in an open question. The answer they gave was inserted into all 8 items of the need to belong scale (e.g., “I try hard to avoid doing things which could lead other [ethnic group] people to exclude me”; α = .82).

***Feelings about gender and ethnic miscategorizations.*** We also wrote, tested and included items to test the level of threat/negative affect of being miscategorized according to gender (3 items, α = .79) and ethnicity (3 items, α = .70): “I would feel threatened if someone didn’t know whether to call me ‘Sir’ or ‘Madam’ at first sight”/”I would feel threatened if someone didn’t know whether I was a Native or a non-Native in the country where I live” and other similar items. We created one item each about how acceptable it is to be mixed-race or mixed-gender: “It is acceptable to be mixed-race”/”mixed-gender”, and one item each about the threat of being unable to categorize others according to their gender or ethnicity: ”I would dislike not knowing whether someone is a man or a woman”/”I would dislike not knowing someone’s ethnic background at first sight”.

**Results: Separate post hoc tests for relationship between GI and positive affect, for White and non-White participants.**

***Positive affect.*** There had been a covariate effect of binary ethnicity (White, non-White) on the significant effect of gender cluster membership on positive affect towards answered questions. To investigate the differences between clusters per ethnicity, we therefore split our data in two: White and on-White participants, and ran the Tukey HSD post-hoc test separately for each binary ethnic category. Among White participants as well as among non-White participants, we found that participants in cluster 2 (low male and low female identifiers) felt significantly less positive affect than participants in clusters 1 (high male and low female identifiers) and 3 (high male and high female identifiers; all *p*s ≤ .01).


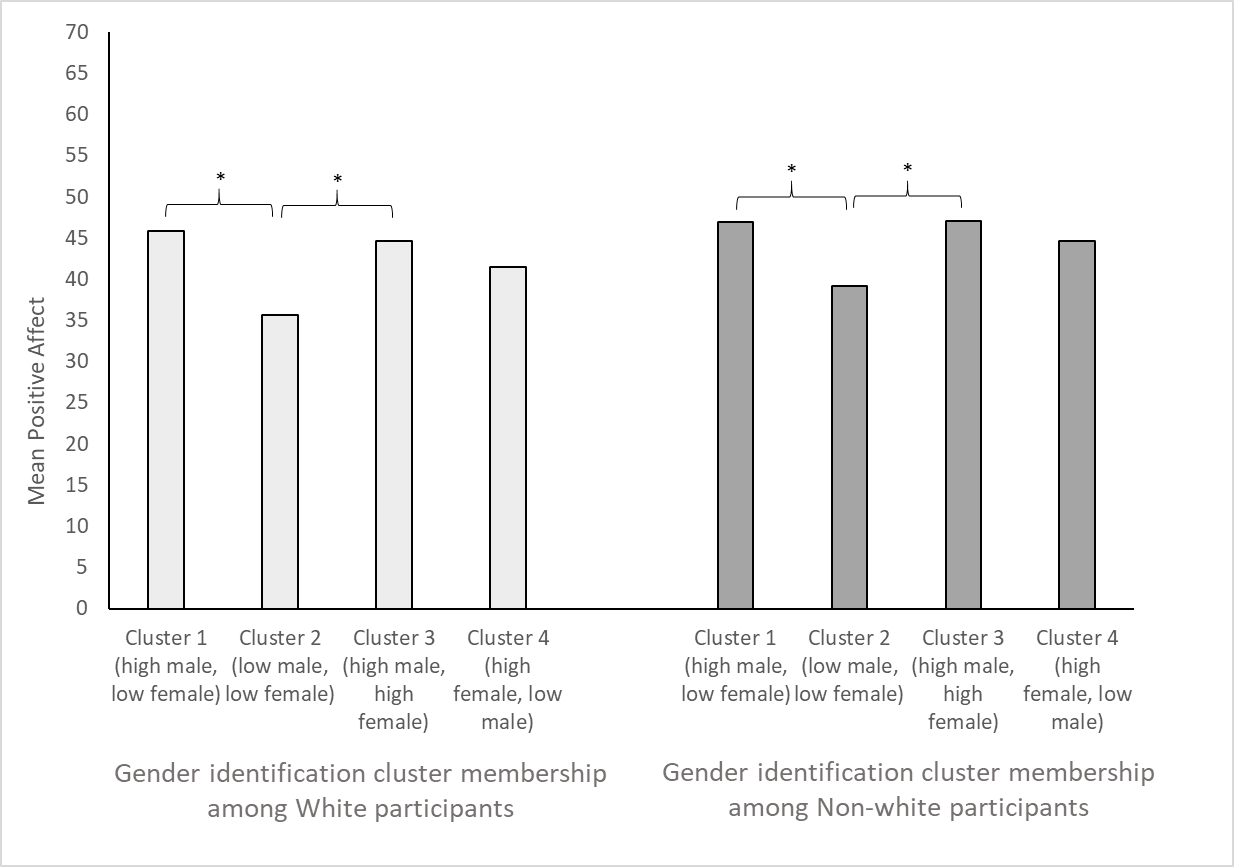


*Figure 1*. Mean positive affect per cluster, per binary ethnicity, in Study 2. Stars (*) denote significant differences between clusters (*p* < .05).

**Results: Beliefs about gender binary and opinions about article content.** To test whether ‘gender-as-binary’ or ‘gender-as-spectrum’ belief was related to GI cluster membership, we ran a chi-square test which revealed a significant difference (*X^2^* = 16.43, *p* < 0.01). A post-hoc z-score test revealed that the differences driving the significant effect were those in cluster 1 and those in cluster 3. We found that people with a high male and low female GI more often believed gender to be binary than a spectrum, and people with a high male and high female GI more often believed gender to be a spectrum rather than binary (see Table 10).

**Table 10**

*Percentages of ‘gender-as-binary’ and ‘gender-as-spectrum’ beliefs in each gender identity cluster, Study 2*

| Belief | Cluster 1 (high male, low female) | Cluster 2 (low male, low female) | Cluster 3 (high male, high female) | Cluster 4 (high female, low male) |
| --- | --- | --- | --- | --- |
| “Gender is binary” | 60.34% | 48.24% | 36.30% | 40.18% |
| “Gender is a spectrum” | 39.66% | 51.76% | 63.70% | 59.82% |

We tested whether GI cluster membership was related to participants’ feelings towards the shown article about non-binary passports in the UK. We ran a MANOVA with GI cluster membership (4 levels) as IV and the following DVs: positive affect towards article, negative affect towards article, defensiveness about issues described in the article, acceptance of issues described in the article, positive responses towards changes in gender narrative described in article and negative responses towards changes in gender narrative described in article.

We found a main effect of gender identification on all dependent variables: positive affect, *F*(3,478) = 5.42, *p* < .01, η**_p_**^2^ = 0.03; negative affect, *F*(3,478) = 4.78, *p* < .01, η**_p_**^2^ = 0.03; defensiveness, *F*(3,478) = 5.00, *p* < .01, η**_p_**^2^ = 0.03; acceptance, *F*(3,478) = 6.74, *p* < .01, η**_p_**^2^ = 0.04; positive responses *F*(3,478) = 5.33, *p* < .01, η**_p_**^2^ = 0.03; and negative responses, *F*(3,478) = 8.31, *p* < .01, η**_p_**^2^ = 0.05.

Given that we had found a main effect of gender cluster membership on all measures of opinions about the article, and for simplification of results for the reader, we ran a higher order factor analysis (oblimin) to pool together positive measures (acceptance, positive opinions and positive affect) into one factor, and negative measures (defensiveness, negative opinions and negative affect) into another factor. Positive measures loaded onto a single factor which explained 72.12% of the variance; negative measures loaded onto a factor with 68.58% of variance explained. We then saved the regression factor score for each factor, which we report upon henceforth.^10^

***Positive measures regarding the article.*** A Tukey’s HSD post hoc analysis revealed that people in cluster 1 (high male, low female identifiers) were significantly less positive towards the article than people in cluster 3 (high male, high female) and cluster 4 (high female, low male), but not people in cluster 2 (low male, low female). People in cluster 2 were significantly less positive towards the article than people in clusters 3 and 4 (all *p*s ≤ .04). Clusters 3 and 4 did not differ significantly in terms of positivity towards the article (see Figure 2).

*Figure 2.* Overall positivity towards article, as measured by the regression score of the factor analysis including acceptance, positive emotions and positive opinions, per cluster, in Study 2. Stars (*) denote significant differences between clusters (*p* < .05). The zero line on the y axis denotes the mean of all groups. A positive result (above x axis) thereby means a positivity score higher than the mean, while a negative result (below x axis) means positivity score lower than the mean.

***Negative measures regarding the article.*** A Tukey’s HSD post hoc analysis revealed that people in cluster 1 (high male, low female identifiers) were significantly more negative towards the article than people in cluster 4 (high female, low male). People in cluster 3 (high male, high female) were significantly more negative towards the article than people in cluster 4 (all *p*s ≤ .02). There were no other significant differences in negativity towards the article (see Figure 3).

*Figure 3.* Overall negativity towards article, as measured by the regression score of the factor analysis including defensiveness, negative emotions and negative opinions, per cluster, in Study 2. Stars (*) denote significant differences between clusters (*p* < .05). The zero line on the y axis denotes the mean of all groups. A positive result (above x axis) thereby means a positivity score higher than the mean, while a negative result (below x axis) means positivity score lower than the mean.

**Results: Separate post hoc tests for relationship between GI and opinions about shown article.**

***Positive affect towards reading the article.*** The Tukey HSD test indicated that people in cluster 2 (low male, low female identifiers) felt significantly less positive affect than people in cluster 3 (high male, high female identifiers) and cluster 4 (high female, low male identifiers) (all *p*s ≤ .01). People in cluster 1 (high male, low female identifiers) did not differ significantly from any of the other groups in terms of how positively they felt towards reading the article.


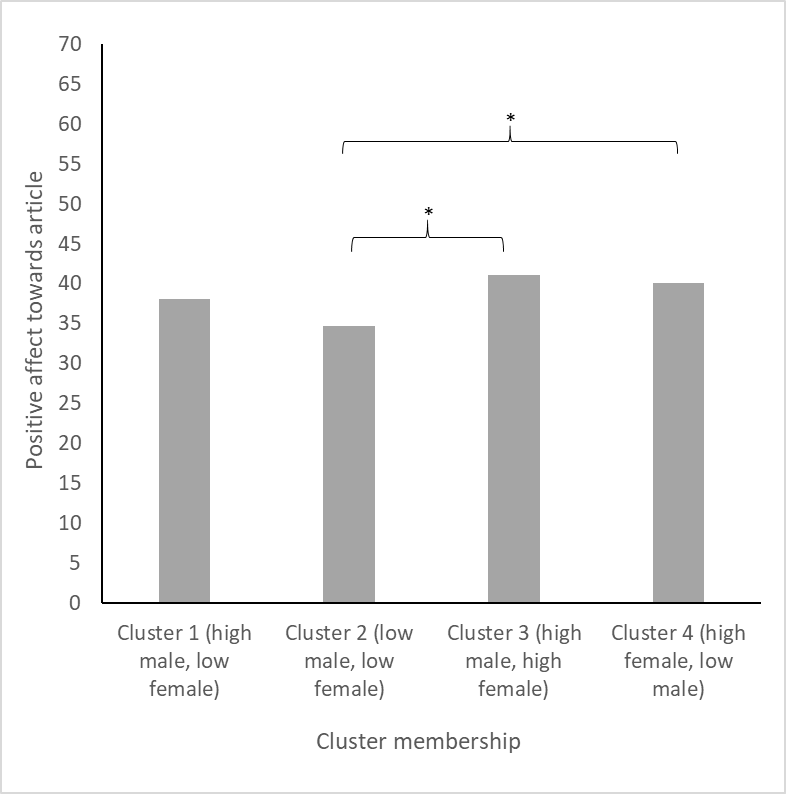


*Figure 4*. Mean positive affect towards reading the article per cluster, Study 2. Stars (*) denote significant differences between clusters (*p* < .05).

***Negative affect towards reading the article.*** The Tukey HSD test revealed that people in cluster 2 (low male, low female identifiers) felt significantly less negative affect than people in cluster 1 (high male, low female identifiers) and cluster 3 (high male, high female identifiers) (all *p*s ≤ .02). People in cluster 4 did not differ significantly from any of the other groups in terms of how negative they felt towards reading the article.


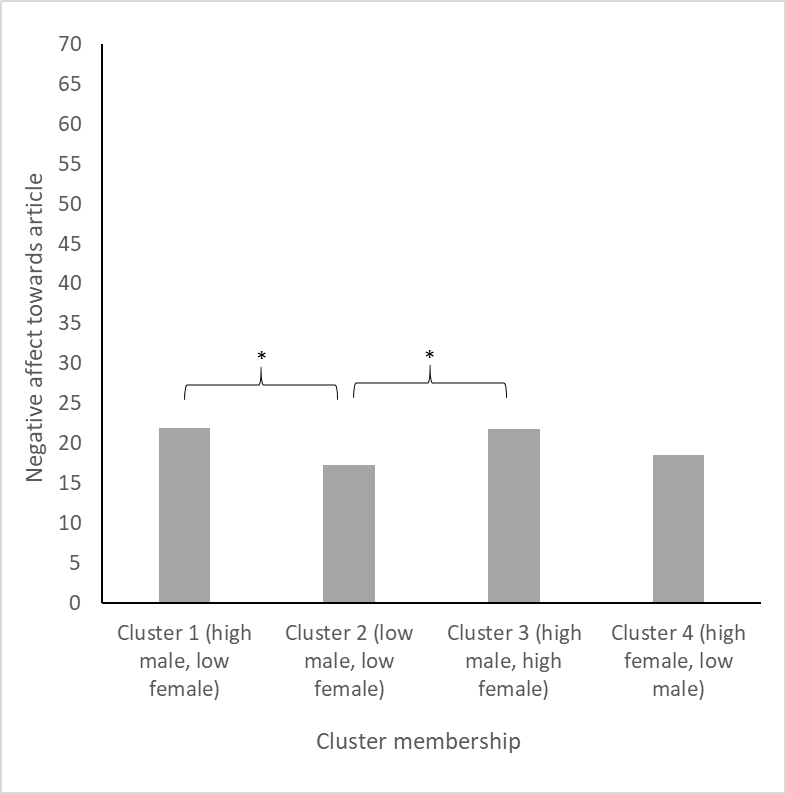


*Figure 5*. Mean negative affect towards reading the article per cluster, Study 2. Stars (*) denote significant differences between clusters (*p* < .05).

***Defensiveness towards issues described in article.*** The Tukey HSD test indicated that people in cluster 1 (high male, low female identifiers), as well as cluster 2 (low male, low female identifiers), responded significantly more defensively towards the issues described in the article than people in cluster 4 (high female, low male identifiers) (all *p*s ≤ .02). People in cluster 3 did not differ significantly from any of the other groups in terms of defensiveness.


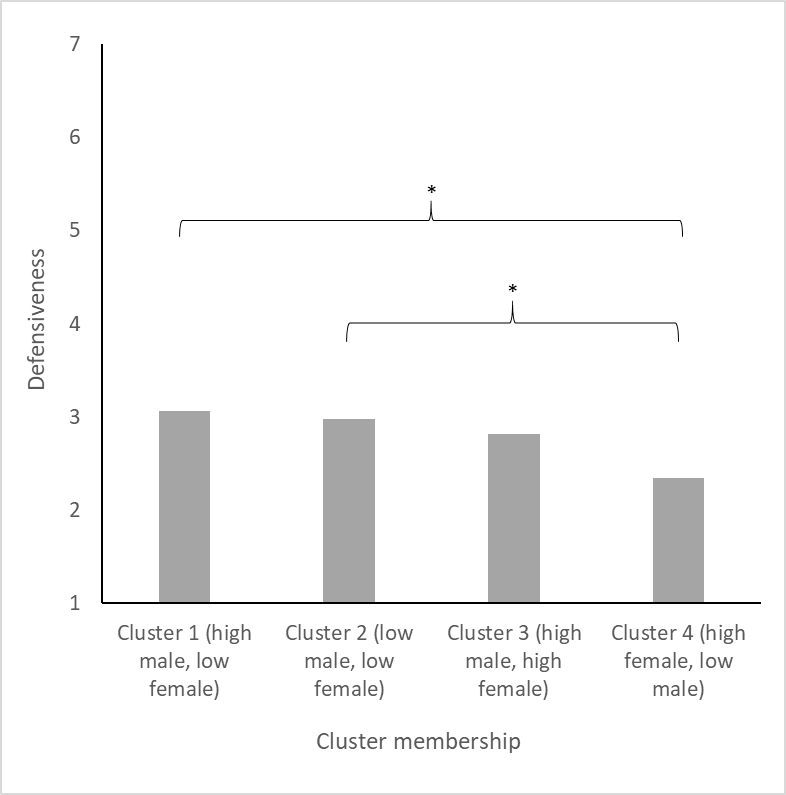


*Figure 6*. Mean defensiveness towards the article per cluster, Study 2. Stars (*) denote significant differences between clusters (*p* < .05).

***Acceptance of issues described in article.*** Tukey’s HSD revealed that people in cluster 1 (high male, low female identifiers) responded significantly less accepting of the issues described in the article than people in cluster 3 (high male, high female identifiers) and cluster 4 (high female, low male identifiers) (all *p*s ≤ 0.01). People in cluster 2 (low male, low female identifiers) did not differ significantly from any of the other groups.


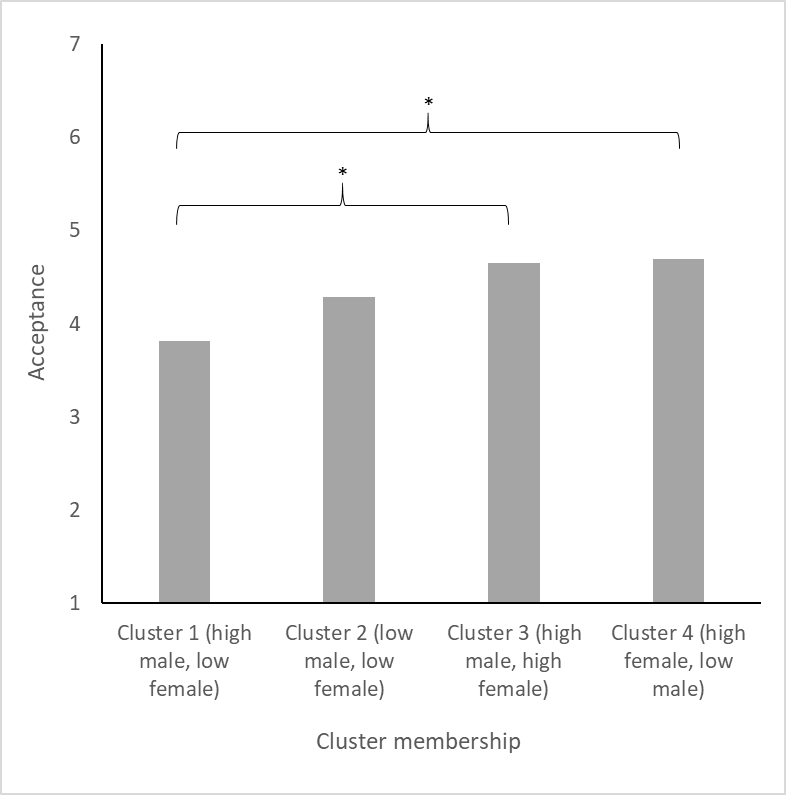


*Figure 7*. Mean acceptance towards the article per cluster, Study 2. Stars (*) denote significant differences between clusters (*p* < .05).

***Positive responses towards changes in gender narrative described in article.*** The post-hoc Tukey HSD indicated that people in cluster 4 (high female, low male identifiers) were significantly more positive in their responses to the changes in the gender narrative described in the article, than people in cluster 1 (high male, low female identifiers) and cluster 2 (low male, low female identifiers) (all *p*s ≤ .03). People in cluster 3 (high male, high female identifiers) did not differ significantly from the other groups in terms of positive responses.


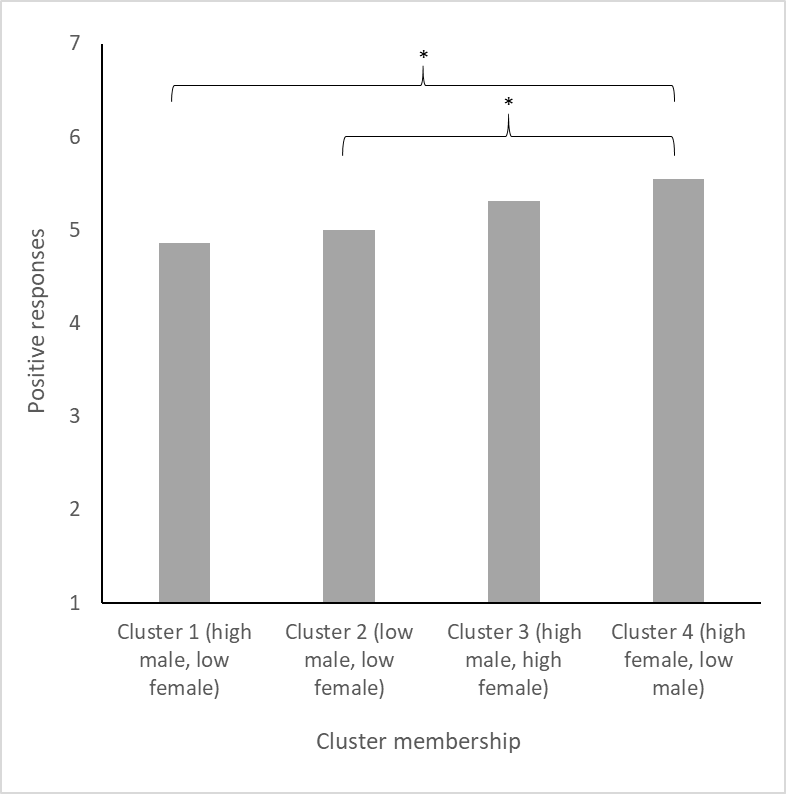


*Figure 8*. Mean positive opinions towards the article per cluster, Study 2. Stars (*) denote significant differences between clusters (*p* < .05).

***Negative responses towards changes in gender narrative described in article.*** The Tukey HSD revealed that people in cluster 1 (high male, low female identifiers) responded significantly more negatively towards the changes in the gender narrative described in the article than people in cluster 3 (high male, high female identifiers) and people in cluster 4 (high female, low male identifiers) (all *p*s ≤ .03). People in cluster 2 (low male, low female identifiers) did not differ significantly from the other groups in terms of negative responses.


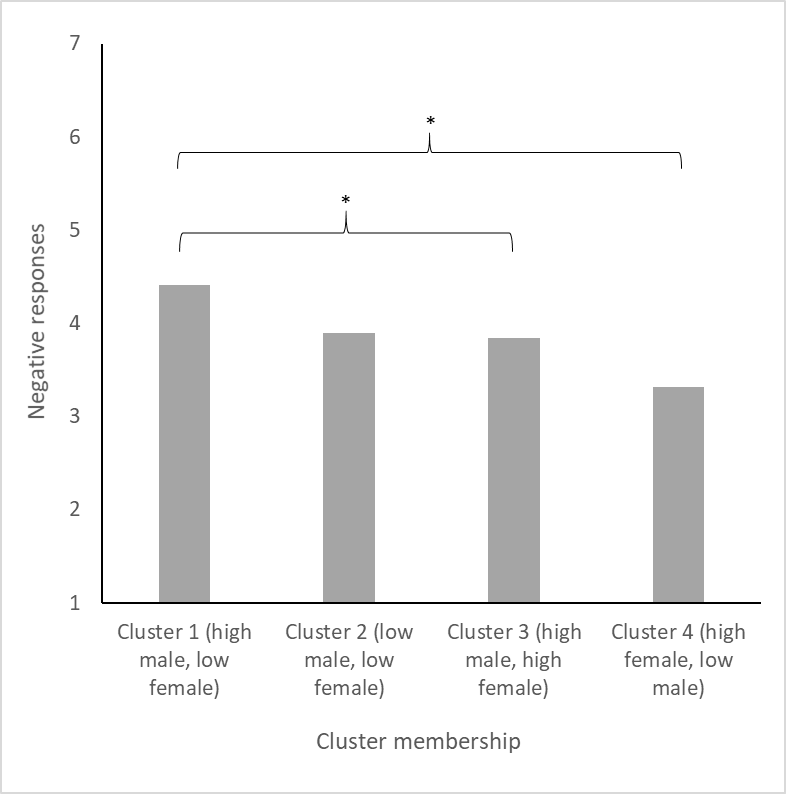


*Figure 9*. Mean negative opinions towards the article per cluster, Study 2. Stars (*) denote significant differences between clusters (*p* < .05).

**Results: other components of GI.** Replicating Study 1, a confirmatory factor analysis with two factors including all cognitive, emotional and evaluative measures of GI, revealed that 10 out of 22 binary-gendered items (half of which are about men, the other half about women) loaded onto separate male and female factors. In contrast to Study 1, the items that loaded onto separate factors were solely the cognitive, rather than the cognitive and emotional identification items.

We can only speculate why we found these different results between our two samples. As Ellemers, Kortekaas and Ouwerkerk (1999) showed, cognitive identification is affected by relative group size while emotional identification is affected by group status and group assignment criterion. One assumption we could therefore make is that participants in Study 1 and Study 2 were similarly affected by relative group size, but differently affected by group status and group assignment criterion.

***Results: Cognitive GI factor loadings.***

**Table 11**

*Factor loadings of each cognitive GIW item for cognitive, emotional and evaluative components, Study 2*

|  | Component 1 (cognitive) | Component 2  (emotional) | Component 3 (evaluative) |
| --- | --- | --- | --- |
| “I feel involved with (other) women” |  | .85 |  |
| “I feel closely connected with (other) women” | .34 | .78 |  |
| “I identify with (other) women” ^a^ | .87 |  |  |
| “I am like (other) women” ^a^ | .79 |  |  |
| “Women are an important reflection of who I am” ^a^ | .78 |  |  |
| “I see myself as someone belonging to the group of women” ^a^ | .86 |  |  |
| “I have a lot in common with (other)  women” ^a^ | .81 |  |  |

*Note.* Only factor loadings > .3 are shown.

^a^ Ultimately included in cognitive identification scale.

**Table 12**

*Factor loadings of each cognitive GIM item for cognitive, emotional and evaluative components, Study 2*

|  | Component 1 (cognitive) | Component 2  (emotional) | Component 3 (evaluative) |
| --- | --- | --- | --- |
| “I feel involved with (other) men” |  | .81 |  |
| “I feel closely connected with (other) men” | .36 | .77 |  |
| “I identify with (other) men” ^a^ | .81 |  |  |
| “I am like (other) men” ^a^ | .80 |  |  |
| “Men are an important reflection of who I am” ^a^ | .76 |  |  |
| “I see myself as someone belonging to the group of men” ^a^ | .82 |  |  |
| “I have a lot in common with (other) men” ^a^ | .77 |  |  |

*Note.* Only factor loadings > .3 are shown.

^a^ Ultimately included in cognitive identification scale.

**Results: Differences between the social constructs of ethnicity and gender.**

We divided participants into two groups in terms of ethnicity: White and non-White. This was a) to keep the construct of ethnicity similar as the construct of gender in our analyses (i.e., it is mostly defined in a binary way) and b) to test differences between having a higher status ethnicity (White) and a lower status ethnicity (all other ethnicities).

A chi-square test using gender cluster membership as DV and ethnicity (binary: White and non-White) as IV revealed no significant differences in cluster memberships across White and non-White ethnicities (*X^2^* = 0.49, *p* = .92). This suggests that ethnic status (high: White, low: non-White) does not affect people’s self-reported GI.

**Table 13**

*Percentages of White and non-White participants in each gender identity cluster, in Study 2*

| Ethnicity | Cluster 1 (high male, low female) | Cluster 2 (low male, low female) | Cluster 3 (high male, high female) | Cluster 4 (high female, low male) |
| --- | --- | --- | --- | --- |
| White | 24.28% | 20.16% | 31.28% | 24.27% |
| Non-White | 26.36% | 19.67% | 28.87% | 25.10% |

To explore the rigidity of the gender binary, as compared to another stigmatized social categorization (ethnicity), we wanted to investigate whether people feel a higher need to belong to their ethnic group or their gender group, and whether this depends on group membership. One might expect that non-White people, due to being marginalized, would feel a higher need to belong to their ethnic group, for example. We therefore ran a 2 (gender assigned at birth, male or female) x 2 (ethnicity coded as White or non-White) MANOVA with mean need to belong to one’s gender group and mean need to belong to one’s ethnic group as dependent variables. We found that need to belong to one’s gender group differed depending on gender assigned at birth (*F*(1,478) = 26.80, *p* < .01, η**_p_**^2^ = 0.05) with women feeling a higher need to belong to the group of women (*M* = 4.03, *SD* = 1.20) than men’s need to belong to the group of men (*M* = 3.50, *SD* = 1.04). We found no main effect of ethnicity, and no interaction effect of ethnicity and gender assigned at birth (*p*s ≥ .84).

A post-hoc paired samples t-test revealed that across the whole sample there was a higher need to belong to one’s gender group than one’s ethnic group (*t*(481) = 6.09, *p* < .01, Cohen’s *d* = 0.24, *M1* = 3.76, *SD1* = 1.15, *M2* = 3.48, *SD2* = 1.18).

We ran a number of paired samples t-tests to find out whether acceptability of being mixed, threat of not categorizing somebody, or threat of being miscategorized by others differed for ethnicity and gender. Our tests revealed that people felt that it is more acceptable to be mixed-race than to be mixed-gender (*t*(481) = 14.13, *p* < .01, Cohen’s *d* = 0.81, *M1* = 6.63, *SD1* = 0.90, *M2* = 5.34, *SD2* = 2.07); people would dislike it more to not know somebody’s gender than to not know somebody’s ethnicity (*t*(481) = 14.77, *p* < .01, Cohen’s *d* = 0.78, *M1* = 3.42, *SD1* = 2.09, *M2* = 2.02, *SD2* = 1.44); people indicated that they would find it more threatening to be miscategorised according to gender than according to ethnicity (*t*(481) = 20.02, *p* < .01, Cohen’s *d* = 1.06, *M1* = 4.02, *SD1* = 1.81, *M2* = 2.34, *SD2* = 1.31).

***Conclusions.*** Although being of a lower status group in one domain (ethnicity) may motivate individuals to identify with higher status groups in another domain (gender), we did not find evidence for that in Study 2.

We found that participants felt that being mixed-gender was less acceptable and more threatening than being mixed-race, and that being miscategorized in terms of one’s gender is a more negative experience than being miscategorized in terms of one’s ethnicity. Participants also reported higher need to belong to their gender group than their ethnic group. This suggests that the rigid binary construction of gender causes people to feel even more negative about gender ambivalence than ethnic ambivalence.

We also explored the extent to which participants thought gender ambivalence is not acceptable, or negative, in Study 2. To do so, we compared participants’ responses to their feelings about being mixed-race, another stigmatized dual identification (Wilton et al., 2013). We found that participants felt that being mixed-gender was less acceptable and more threatening than being mixed-race, and that being miscategorized in terms of one’s gender is a more negative experience than being miscategorized in terms of one’s ethnicity. Participants also reported higher need to belong to their gender group than their ethnic group. This suggests that the rigid binary construction of gender causes people to feel even more negative about gender ambivalence than ethnic ambivalence.

**Study 3**

**Participant demographics.** For more information about participants’ education level, employment, disabilities and country of residence please see tables below.

**Table 14**

*Percentage of participants in Study 3 per education level*

| Education level | Percentage |
| --- | --- |
| Less than high school | 1.42% |
| High school graduate | 23.13% |
| Some college/university | 22.42% |
| Bachelor degree | 32.74% |
| Master degree | 19.22% |
| Doctorate | 1.07% |

**Table 15**

*Percentage of participants in Study 3 per employment situation*

| Employment Situation | Percentage |
| --- | --- |
| Employed full time | 36.30% |
| Employed part time | 10.68% |
| Unemployed looking for work | 13.88% |
| Unemployed not looking for work | 3.91% |
| Retired | 0.71% |
| Student | 33.45% |
| Unemployed due to disability | 1.07% |

**Table 16**

*Percentage of participants in Study 3 per special needs situation*

| Disability | Percentage |
| --- | --- |
| Physical | 3.56% |
| Mental | 8.19% |
| Both | 1.42% |
| Neither | 86.83% |

**Table 17**

*Percentage of participants in Study 3 per country of residence*

| Country of residence | Percentage |
| --- | --- |
| Austria  Belgium  Canada  Chile  Czech Republic  Estonia  Finland  France  Germany  Greece  Hungary  Ireland  Israel  Italy  Latvia  Mexico  Netherlands  Norway  Poland  Portugal  South Africa  Spain  Sweden  United Kingdom  United States | 0.36%  0.71%  0.71%  1.43%  0.71%  0.71%  0.71%  2.85%  1.78%  2.14%  1.07%  0.36%  1.07%  11.74%  0.71%  3.91%  1.78%  0.36%  14.23%  18.51%  1.78%  4.63%  0.71%  20.28%  3.56% |

**Extra measures: Precarious Manhood and Womanhood.** In order to measure beliefs about how precarious of a state manhood and womanhood are, we administered 14 items (seven about men, seven about women) which include “Manhood is something that can be taken away” (Vandello, Bosson, Cohen, Burnaford & Weaver, 2008; items about men α = .83, items about women α = .81).

**Results: two-way cluster analysis.**

For replication purposes, we ran a series of cluster analyses with two inputs (GIM and GIW), and found that a 3-cluster model (compared to 2, 4, 5, and 6-cluster models) was the best fit in terms of percentage of variance, Bayesian Information Criterion (*BIC* = 143,41) and silhouette of cohesion (Silhouette ≥ .5) and separation combined. See Table 18 for cluster centres and percentages of sample in each cluster. See Figure 10 for more information about participant responses on the two binary gender measures.

**Table 18**

*Cluster centres for GIW and GIM and percentage of participants who fell into each cluster in two-way model, Study 3*

|  | Cluster 1 (high female, low male) | Cluster 2 (medium female and male) | Cluster 3 (low female, high male) |
| --- | --- | --- | --- |
| Cluster centre for GIW | 6.68 | 5.36 | 2.45 |
| Cluster centre for GIM | 1.65 | 3.29 | 5.84 |
| Percentage of overall sample | 27.40% | 24.55% | 48.04% |

We also ran a series of cluster analyses with the two binary Overlap of Self and Group measures (Overlap of Self and Women, Overlap of Self and Men). Given the nature of this measure being one item measured on a 7-point Likert scale, we had to do a categorical cluster analysis, since single items cannot be treated as continuous. We found that a 3-cluster model was the best fit in terms of Bayesian Information Criterion (*BIC* = 1653.04), silhouette of cohesion and separation, and content of the clusters (additional clusters did not show a different pattern from those in the 3-cluster solution; see Table 19). This is further evidence that adding a third gender category seems to shift participants’ responses towards reporting higher identification with binary genders. This finding is not in line with the the consistent findings of four clusters by Martin et al. (2017), Andrews et al. (2019), and Endendijk et al. (2019), as well as our own research, and further research is needed to investigate why this shift may occur.

**Table 19**

*Cluster centres (medians) for OSW and OSM and percentage of participants in each cluster, Study 3*

|  | Cluster 1 (high female, low male) | Cluster 2 (medium female and male) | Cluster 3 (low female, high male) |
| --- | --- | --- | --- |
| Cluster centre for OSW | 7 | 4 | 2 |
| Cluster centre for OSM | 2 | 5 | 7 |
| Percentage of overall sample | 41.43% | 41.07% | 17.50% |

*Figure 10*. Scatter plot of GIM (Gender Identification with Men) and GIW (Gender Identification with Women), Study 3. Triangles represent AMAB participants, circles represent AFAB participants.

**Results: Relationship between GI and beliefs of precarious manhood and womanhood.**

To test whether participants in different GI clusters had different beliefs about the precariousness of manhood and womanhood, we ran a one-way ANOVA with GI cluster membership as IV and mean precarious manhood and mean precarious womanhood beliefs as DVs. We found a significant effect of GI cluster membership on precarious manhood beliefs, *F*(3,277) = 2.92, *p* = 0.03, but no significant effect on precarious womanhood beliefs, *F*(3,277) = 2.48, *p* = 0.06.

A Tukey HSD post-hoc revealed that the significant difference in precarious manhood beliefs was driven by a difference between people in cluster 1 (male identifiers) and people in cluster 4 (female identifiers), such that cluster 1 believed manhood to be more precarious. No other significant differences between clusters were found. Our expectation, that people in gender ambivalent clusters would believe manhood or womanhood to be less precarious than people in gender binary clusters, was thus not met.

**References**

Andrews, N. C., Martin, C. L., Cook, R. E., Field, R. D., & England, D. E. (2019). Exploring

dual gender typicality among young adults in the United States. *International Journal*

*of Behavioral Development*, *43*(4), 314-321.

Bem, S. L. (1974). The measurement of psychological androgyny. *Journal of Consulting and Clinical Psychology*, *42*(2), 155. doi:[10.1037/h0036215](https://psycnet.apa.org/doi/10.1037/h0036215)

Bergami, M., & Bagozzi, R. P. (2000). Self‐categorization, affective commitment and group self‐esteem as distinct aspects of social identity in the organization. *British Journal of Social Psychology*, *39*(4), 555-577. doi:[10.1348/014466600164633](https://doi.org/10.1348/014466600164633)

Burch, B. (1993). Gender identities, lesbianism, and potential space. *Psychoanalytic*

*Psychology*, *10*(3), 359. <https://doi.org/10.1037/h0079454>

Derks, B., Van Laar, C., & Ellemers, N. (2009). Working for the self or working for the group: How self-versus group affirmation affects collective behavior in low-status groups. *Journal of Personality and Social Psychology*, *96*(1), 183. doi:[10.1037/a0013068](https://doi.org/10.1037/a0013068)

Ellemers, N., Kortekaas, P., & Ouwerkerk, J. W. (1999). Self‐categorisation, commitment to

the group and group self‐esteem as related but distinct aspects of social identity.

*European journal of social psychology*, *29*(2‐3), 371-389.

Endendijk, J. J., Andrews, N. C., England, D. E., & Martin, C. L. (2019). Gender-identity

typologies are related to gender-typing, friendships, and social-emotional adjustment in

Dutch emerging adults. *International Journal of Behavioral Development*, *43*(4), 322

333.

Fusion’s Massive Millennial Survey (2015) *Fusion.net*. Retrieved from <http://fusion.net/event/massive-millennial-poll/>

Goldman, B. M., & Kernis, M. H. (2002). The role of authenticity in healthy psychological

functioning and subjective well-being. *Annals of the American Psychotherapy*

*Association*, *5*(6), 18-20.

Henderson-King, D. H., & Stewart, A. J. (1994). Women or feminists? Assessing women's

group consciousness. *Sex Roles*, *31*(9-10), 505-516.

<https://doi.org/10.1007/BF01544276>

Howell, J. L., & Ratliff, K. A. (2014). Implicit-explicit attitude discrepancy prompts defensive responding to IAT feedback. *Unpublished Manuscript. University of Florida*.

Howell, J. L., Redford, L., Pogge, G., & Ratliff, K. A. (2017). Defensive responding to IAT feedback. *Social Cognition*, *35*(5), 520-562. doi:[10.1521/soco.2017.35.5.520](https://doi.org/10.1521/soco.2017.35.5.520)

Kuper, L. E., Nussbaum, R., & Mustanski, B. (2012). Exploring the diversity of gender and

sexual orientation identities in an online sample of transgender individuals. *Journal of*

*sex research*, *49*(2-3), 244-254. <https://doi.org/10.1080/00224499.2011.596954>

Leary, M. R., Kelly, K. M., Cottrell, C. A., & Schreindorfer, L. S. (2013). Construct validity

of the need to belong scale: Mapping the nomological network. *Journal of personality*

*assessment*, *95*(6), 610-624. <https://doi.org/10.1080/00223891.2013.819511>

Martin, C. L., Andrews, N. C., England, D. E., Zosuls, K., & Ruble, D. N. (2017). A dual

identity approach for conceptualizing and measuring children's gender identity. *Child*

*Development*, *88*(1), 167-182. <https://doi.org/10.1111/cdev.12568>

Richard, F. D., Bond, C. F. Jr., & Stokes-Zoota, J. J. (2003). One hundred years of social

psychology quantitatively described. *Review of General Psychology*, *7*(4), 331-363.

<https://doi.org/10.1037/1089-2680.7.4.331>

Schneider, D. (2012). Gender deviance and household work: The role of occupation.

*American Journal of Sociology*, *117*(4), 1029-1072. <https://doi.org/10.1086/662649>

Turner, J. C., & Reynolds, K. J. (1987). A self-categorization theory. *Rediscovering the*

*social group: A self-categorization theory*.

Vandello, J. A., Bosson, J. K., Cohen, D., Burnaford, R. M., & Weaver, J. R. (2008).

Precarious manhood. *Journal of personality and social psychology*, *95*(6), 1325.

Watson, D., Clark, L. A., & Tellegen, A. (1988). Development and validation of brief

measures of positive and negative affect: the PANAS scales. *Journal of personality*

*and social psychology*, *54*(6), 1063.

Wilton, L. S., Sanchez, D. T., & Garcia, J. A. (2013). The stigma of privilege: Racial identity

and stigma consciousness among biracial individuals. *Race and Social Problems*,

*5*(1), 41-56. <https://doi.org/10.1007/s12552-012-9083-5>

**Appendix A**

**Cognitive Gender Identification (final items used in main manuscript, Studies 1 & 2)**

I identify with (other) women

I identify with (other) men

I am like (other) women

I am like (other) men

Women are an important reflection of who I am

Men are an important reflection of who I am

I see myself as someone belonging to the group of women

I see myself as someone belonging to the group of men

I have a lot in common with (other) women

I have a lot in common with (other) men

I feel involved with (other) women *(not used in Study 2)*

I feel involved with (other) men *(not used in Study 2)*

I feel closely connected with (other) women *(not used in Study 2)*

I feel closely connected with (other) men *(not used in Study 2)*

**Emotional Gender Identification**

In another life, I feel that I would enjoy being a woman

In another life, I feel that I would enjoy being a man

I would dislike being a woman (R)

I would dislike being a man (R)

I would rather be a woman than a man

I would rather be a man than a woman

**Evaluative Gender Identification**

I like (other) women

I like (other) men

I have little respect for (other) women (R)

I have little respect for (other) men (R)

I think women have little to be proud of (R)

I think men have little to be proud of (R)

**Appendix B**
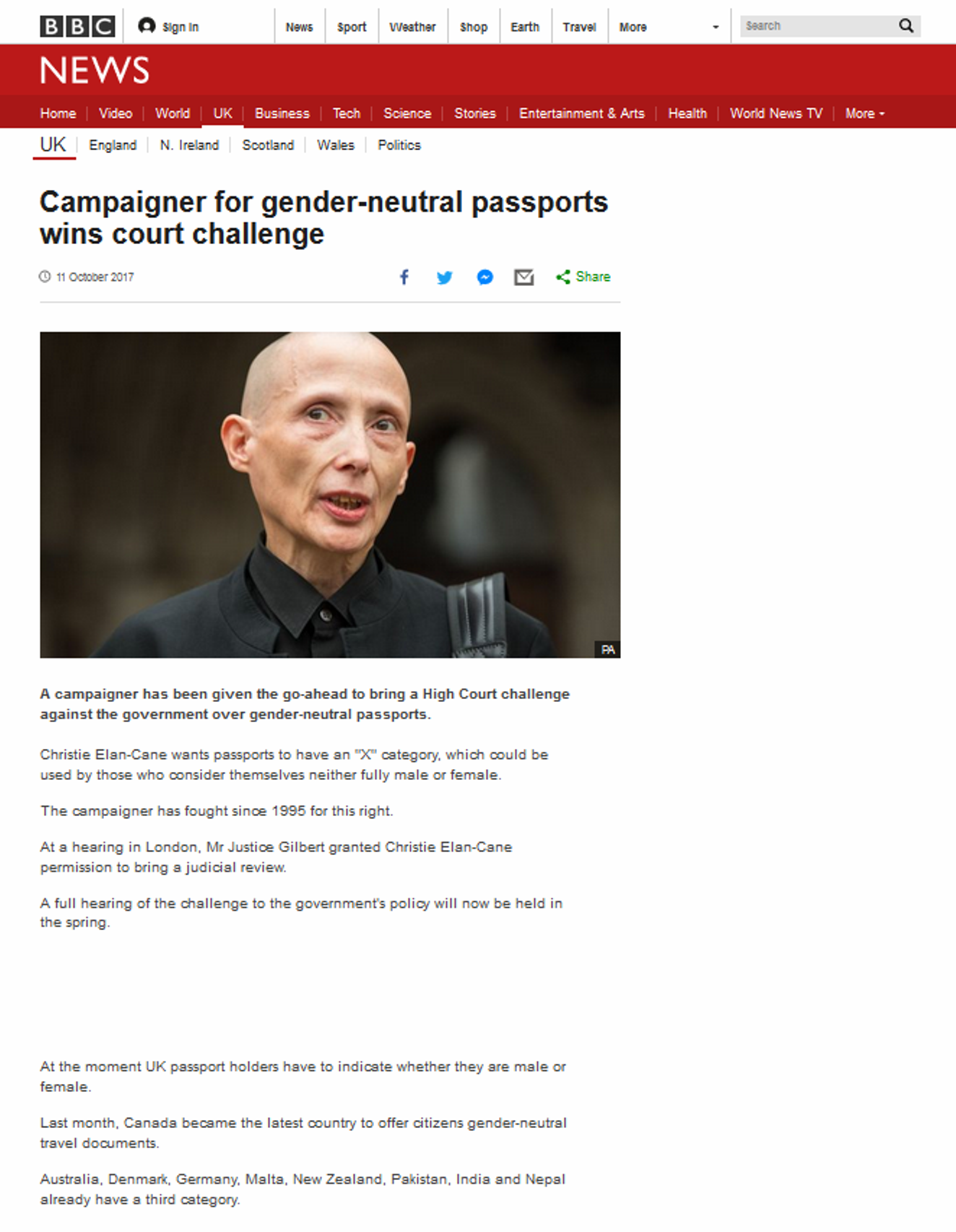


*Figure 11*. BBC article about legal recognition of non-binary genders in the UK, shown to participants of Study 2.

**Appendix C**

**Positive and negative opinions about shown article: Questionnaire items.**

***Positive items.***

“I think it is good that action is taken within societies to be more inclusive of non-binary genders.”

“I think it is very important that the structural disadvantaging of non-binary individuals is being countered.”

“I think it’s important that societies treat non-binary people fairly and justly.”

***Negative items.***

“Countering the disadvantage of non-binary people is not my problem.”

“I do not believe that the societal exclusion of non-binary individuals is a real problem and societies should not pay much attention to it.”

“Societies should make the exclusion of non-binary people less prominent to the public.”

“I would like to forget about the disadvantages of non-binary people and what we are doing to be more inclusive of them.”

“I would rather not get mixed up in discussions about the existence and problems of non-binary people.”

“Non-binary people are complainers and there are other problems societies should focus on.”

“Non-binary people are oversensitive and/or are simply seeking attention.”
